# Menarche, menopause, and breast cancer risk: individual participant meta-analysis, including 118 964 women with breast cancer from 117 epidemiological studies

**DOI:** 10.1016/S1470-2045(12)70425-4

**Published:** 2012-11

**Authors:** Collaborative Group on Hormonal Factors in Breast Cancer

## Abstract

**Background:**

Menarche and menopause mark the onset and cessation, respectively, of ovarian activity associated with reproduction, and affect breast cancer risk. Our aim was to assess the strengths of their effects and determine whether they depend on characteristics of the tumours or the affected women.

**Methods:**

Individual data from 117 epidemiological studies, including 118 964 women with invasive breast cancer and 306 091 without the disease, none of whom had used menopausal hormone therapy, were included in the analyses. We calculated adjusted relative risks (RRs) associated with menarche and menopause for breast cancer overall, and by tumour histology and by oestrogen receptor expression.

**Findings:**

Breast cancer risk increased by a factor of 1·050 (95% CI 1·044–1·057; p<0·0001) for every year younger at menarche, and independently by a smaller amount (1·029, 1·025–1·032; p<0·0001), for every year older at menopause. Premenopausal women had a greater risk of breast cancer than postmenopausal women of an identical age (RR at age 45–54 years 1·43, 1·33–1·52, p<0·001). All three of these associations were attenuated by increasing adiposity among postmenopausal women, but did not vary materially by women's year of birth, ethnic origin, childbearing history, smoking, alcohol consumption, or hormonal contraceptive use. All three associations were stronger for lobular than for ductal tumours (p<0·006 for each comparison). The effect of menopause in women of an identical age and trends by age at menopause were stronger for oestrogen receptor-positive disease than for oestrogen receptor-negative disease (p<0·01 for both comparisons).

**Interpretation:**

The effects of menarche and menopause on breast cancer risk might not be acting merely by lengthening women's total number of reproductive years. Endogenous ovarian hormones are more relevant for oestrogen receptor-positive disease than for oestrogen receptor-negative disease and for lobular than for ductal tumours.

**Funding:**

Cancer Research UK.

## Introduction

Menarche and menopause are markers of onset and cessation, respectively, of ovarian and related endocrine activity associated with reproduction. During women's reproductive years (broadly the time between menarche and menopause) the ovary produces steroid hormones that directly affect development and function of the breast. Early menarche and late menopause are known to increase women's risk of developing breast cancer. To assess reliably the strengths of these associations and whether they vary by tumour subtype or by characteristics of affected women requires large numbers, and we address these questions by combining information from more than 100 epidemiological studies. Combining individual participant data from many studies not only increases statistical power but also permits similar definitions and similar analytical methods to be used across studies.

## Methods

### Search strategy and selection criteria

This collaboration began in 1992, and has published on breast cancer risk associated with use of hormonal therapies and childbearing practices.^1^, ^2^, ^3^, ^4^ Potentially eligible epidemiological studies have been sought at regular intervals by computer-aided literature searches, by written communication and discussions with colleagues, and by discussions at scientific meetings, including collaborators' meetings in Oxford in 1993, 1995, 2000, 2005, and 2011 (appendix p 3 shows search strategy and selection criteria). Principal investigators of eligible studies were invited to join the collaboration.

### Data extraction

Cases were women with invasive breast cancer and controls were women without breast cancer. So that similar analytical methods could be used across studies, we incorporated cohort studies using a nested case–control design, in which up to four controls were selected at random, matched at follow-up to age of the case at cancer diagnosis and, where appropriate, by broad geographical region. Data for a range of sociodemographic, reproductive, and other behavioural factors, covering the time period to onset of disease for cases and to an equivalent time for controls, were sought from principal investigators (appendix p 3).

We included studies in these analyses if individual data had been provided for women's menopausal status, age at menarche and, if appropriate, age at menopause, and whether or not they had had a hysterectomy or a bilateral oophorectomy. Women who had had a natural menopause or who had had a bilateral oophorectomy before their natural menopause were classified as postmenopausal, but those who had had a hysterectomy without bilateral oophorectomy before their natural menopause were classified as being of unknown menopausal status (because hysterectomy can mask cessation of ovarian activity). Otherwise, we took definitions used by principal investigators to classify each woman by her age at menarche, menopausal status and, for postmenopasual women, by her age at menopause. Women with unknown menopausal status or unknown ages at menarche or menopause were excluded from analyses, as were women who had used menopausal hormone therapy, since such use can mask the onset of menopause and modify associations between hormonal factors and breast cancer risk.^1^

We also sought information about tumour characteristics—ie, about oestrogen receptor status and about tumour histology. We used information provided by principal investigators to classify tumours as oestrogen receptor-positive or oestrogen receptor-negative, and as ductal, lobular, or of other histology.

### Statistical analysis

We did all analyses using conditional logistic regression, similar in principle to the Mantel-Haenszel stratification technique used in previous reports from this collaboration.^1^, ^2^, ^3^, ^4^, ^5^, ^6^ When two groups were compared odds ratios (ORs, described as relative risks [RRs] when cases and controls are compared) and standard CIs are given. When more than two groups were compared, we estimated variances for every group, treating the ORs or RRs as floating absolute risks,^7^ because this method enables valid comparisons between any two groups, even if neither is the baseline group. This method does not alter risk estimates, but yields variances for each non-baseline group that are slightly smaller than the variances calculated with conventional methods (because these include the variance of the baseline group) and we used these variances to calculate group-specific CIs. Any comparison between two risk estimates must take the variation in each group into account.

Analyses of the association between various factors and women's age at menarche and age at menopause were restricted to controls, and we calculated ORs stratifying by study, by centre within study, and where appropriate by age at diagnosis (≤20 years, and then in 3 year age groups, 21–23 years to 87–89 years); by year of birth (<1920, 1920–29, 1930–39, 1940–49, and ≥1950); by parity and age when first child was born (nulliparous women were a separate stratum and parous women were cross-classified by parity [1–2, ≥3] and age at first birth [<20 years, 20–29 years, ≥30 years]); by current body-mass index (BMI; <25 kg/m^2^, 25–29 kg/m^2^, ≥30 kg/m^2^); by height (<160 cm, 160–164 cm, ≥165 cm); by smoking (never, past, present); and by alcohol consumption per week (<50 g and ≥50 g). Women with unknown values for any adjustment variable were assigned to separate strata.

Analyses of the RR of breast cancer were routinely stratified by the same factors as described above. The effects on the main findings of other potential confounding factors (ethnic origin, hormonal contraceptive use, family history of breast cancer) were also examined. We restricted analyses comparing breast cancer risk in premenopausal, perimenopausal, and postmenopausal women to women aged 45–54 years (since each category is represented in this narrow age band) and we used the same stratifications as described above, except that age was stratified by single years. We stratified analyses relating to tumour characteristics by study, centre within study, age at diagnosis in single years, and year of birth, and adjusted by parity, age at first birth, BMI, smoking, and alcohol consumption, also using the same categories as described previously.

We calculated RRs for breast cancer per 1 year younger at menarche and per 1 year older at menopause by linear regression, using the mean age within each category. We made comparisons across different subgroups of women using standard χ^2^ tests for heterogeneity, calculated from the change in log likelihood on addition of extra terms. Significance tests for heterogeneity by tumour subtype were based on analyses within cases only (because controls provide no additional information), stratified by age, study, and BMI and adjusted for other variables listed previously. We did our analyses with STATA (version 11).

When results for large numbers of subgroups are presented in the figures 99% CIs (or group-specific 99% CIs) are given, to take account of multiple testing. In the text all CIs quoted are 95% CIs.

### Role of the funding source

The funders had no role in study design, data collection, analysis, or interpretation of data, preparation of the report, or decision to publish. All members of the analysis and writing committee (VB, DB, RP, KP, GR) had access to the raw data and are responsible for the final submission for publication.

## Results

Overall 117 studies, together including 118 964 women with breast cancer (cases) and 306 091 without the disease (controls), were included in these analyses. Appendix pp 4–5 show details of every contributing study, including a reference to each, and study-specific information about the women included in the analyses. The 117 studies were done in 35 countries, mostly in Europe or North America. Median year of birth of women with breast cancer was 1939 (IQR 1930–1948), and median year of cancer diagnosis was 1993 (1986–1998). Median age at cancer diagnosis was 54 years (IQR 44–64).

**Figure 1:**
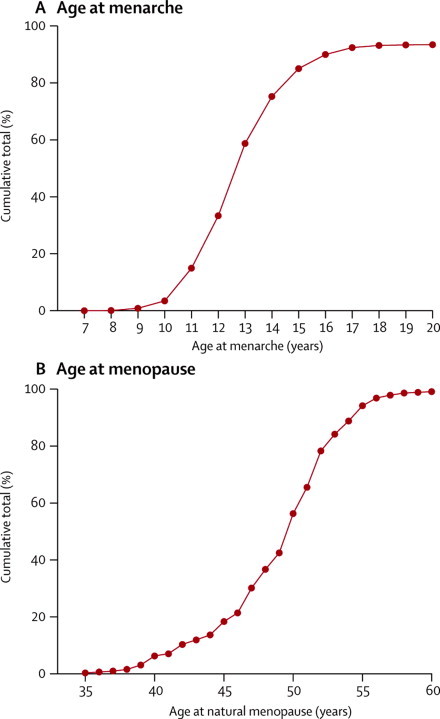
Cumulative distribution of (A) age at menarche and (B) age at natural menopause Data are for women without breast cancer—ie, controls. Results for age at menarche are based on data from 306 068 women. Results for age at menopause are based on data from 157 272 postmenopausal women aged older than 55 years at the time of reporting their age at menopause.

**Figure 2:**
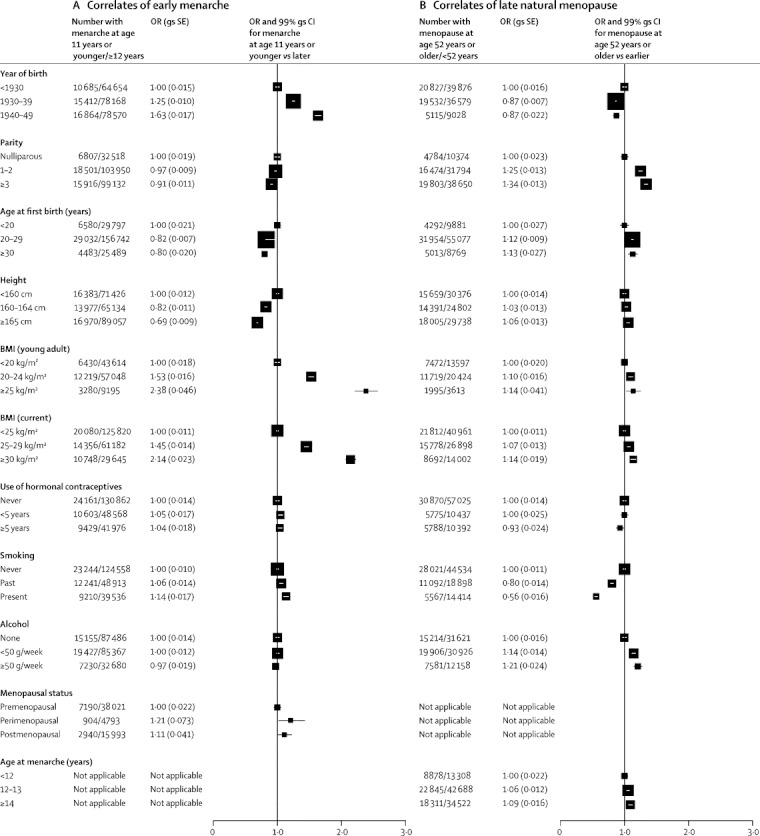
Correlates of various factors with (A) early menarche and (B) late natural menopause Data are for women without breast cancer—ie, controls. OR calculations were stratified by study and, where appropriate, by age at diagnosis, year of birth, parity, age at first birth, smoking, alcohol consumption, height, and current BMI (results for BMI as a young adult are not stratified by current BMI). Results for age at menopause are restricted to postmenopausal women without breast cancer aged 55 years or older at the time they reported their age at menopause. OR=odds ratio. gs=group specific. BMI=body-mass index.

Figure 1A shows the distribution of age at menarche reported by controls—ie, women without breast cancer. Their mean age at menarche was 13·1 years (SD 1·7), with almost two-thirds (65%, 198 113 of 306 068) reporting menarche at ages 12, 13, or 14 years. Only 16% (49 464) of the controls reported menarche at age 11 years or younger and 19% (58 514) reported menarche at age 15 years or older. Having early menarche was associated with many factors known to affect breast cancer risk, including parity, age at first birth, height, and BMI (figure 2A). Increasing adiposity, both as a young adult and currently, showed the strongest associations with early menarche. Associations with late menarche were generally the converse of associations with an early menarche (appendix p 7).

**Figure 3:**
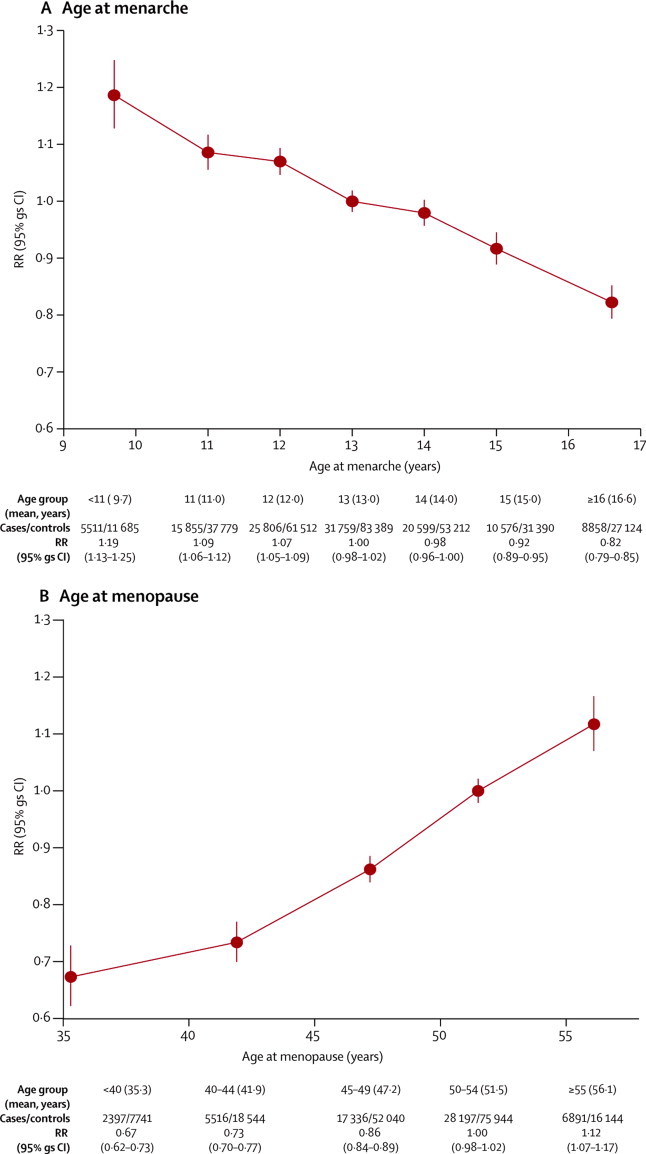
Relative risk of breast cancer by (A) age at menarche and (B) age at menopause Calculated stratifying by study, age, year of birth, parity, age at first birth, smoking, alcohol consumption, height, and current body-mass index. RR=relative risk. gs=group specific.

The younger women were at menarche, the greater was their subsequent risk of breast cancer, the RR increasing by a factor of 1·050 (95% CI 1·044–1·057, p<0·0001) for every year younger at menarche (figure 3A). Results in Figure 3A were stratified by study, age, year of birth, parity and age at first birth, height, current BMI, smoking, and alcohol consumption. Additional adjustment (either individually or simultaneously) by ethnic origin, hormonal contraceptive use, and family history of breast cancer, altered the excess RR estimate by less than 1% (data not shown).

**Figure 4:**
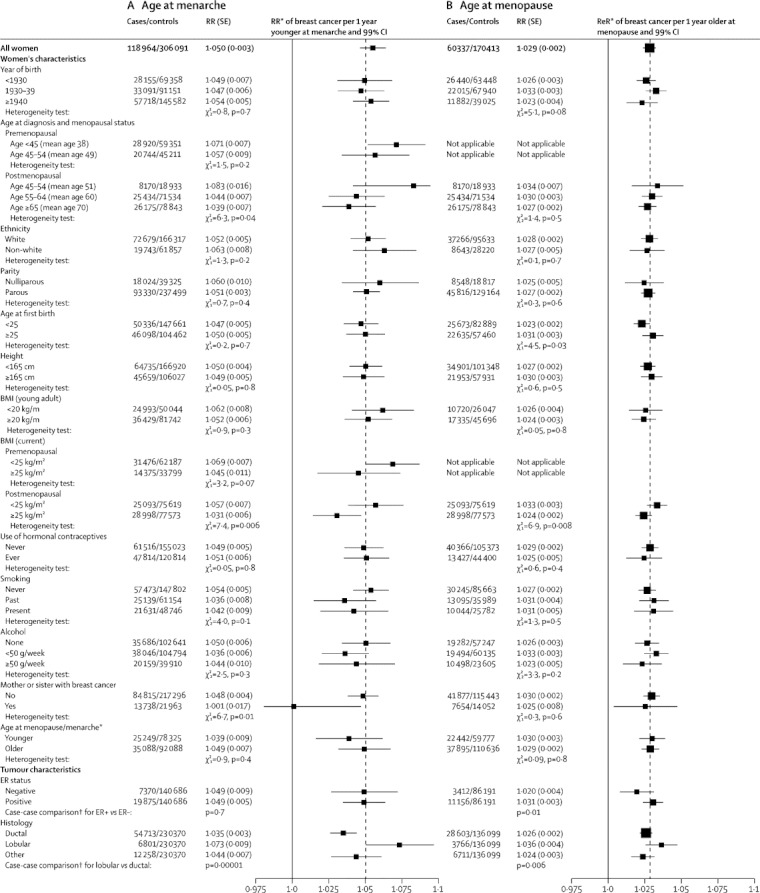
Relative risk of breast cancer (A) per year younger at menarche and (B) per year older at menopause, in various subgroups and by tumour characteristics Relative risks are calculated stratifying by study and age, and where appropriate, by year of birth, parity, age at first birth, smoking, alcohol consumption, height, and current BMI (results for BMI as a young adult are not stratified by current BMI). RR=relative risk. BMI=body-mass index. ER=oestrogen receptor. *Subgroup analyses for age at menarche are for age at menopause <50 *vs* ≥50 years in postmenopausal women; and for age at menopause are for age at menarche <13 *vs* ≥13 years. †Case-case comparisons stratified by study, age and year of birth, and adjusted by parity, age at first birth, smoking, alcohol consumption, height, and current BMI.

To assess consistency of findings, we calculated the RR of breast cancer per year younger at menarche for 34 subgroups of women, subdivided by 14 of their personal characteristics: year of birth, age at diagnosis and menopausal status, ethnic origin, parity, age at first birth, height, BMI as a young adult, current BMI, use of oral contraceptives, smoking, alcohol consumption, family history of breast cancer, menopausal status and, for postmenopausal women, their age at menopause (figure 4A). Both among premenopausal and among postmenopausal women, increasing BMI seemed to attenuate the relevance of age at menarche, but this attenuation was significant only among postmenopausal women (heterogeneity p=0·006). We noted some weakening of the association with age at menarche by attained age in postmenopausal women (heterogeneity p=0·04; trend p=0·02) and family history of breast cancer (heterogeneity p=0·01), but for all other personal characteristics examined heterogeneity across subgroups was not significant. The findings were not dominated by the results in any particular study (appendix pp 8–10) and there was no significant heterogeneity in the findings by study design (appendix p 6).

Of the 117 contributing studies, 85 provided some information about tumour characteristics (appendix pp 4–5), and figure 4A shows the RRs per year younger at menarche for various tumour subtypes. The association with age at menarche was significantly stronger for lobular than ductal tumours (heterogeneity p=0·0001), but there were no significant differences by oestrogen receptor status. Cross-classification by both oestrogen receptor status and tumour histology did not show any further interaction (appendix p 6).

Figure 1B shows the cumulative distribution of the reported age at natural menopause among postmenopausal controls. Their mean age at natural menopause was 49·3 years (SD 4·6), with 15% (26 285 of 170 413) reporting menopause before age 45 years, 75% (127 984) between the ages 45 and 54 years, and 10% (16 144) at age 55 years or older. There was little association between women's reported ages at natural menopause and at menarche (correlation coefficient=0·001). Having a late menopause was weakly associated with women's year of birth, childbearing history, BMI, and alcohol consumption, but the strongest association observed was with smoking, which was negatively associated with a late menopause. (figure 2B). Associations with an early menopause were generally the converse of those associated with a late menopause (appendix p 7).

**Figure 5:**
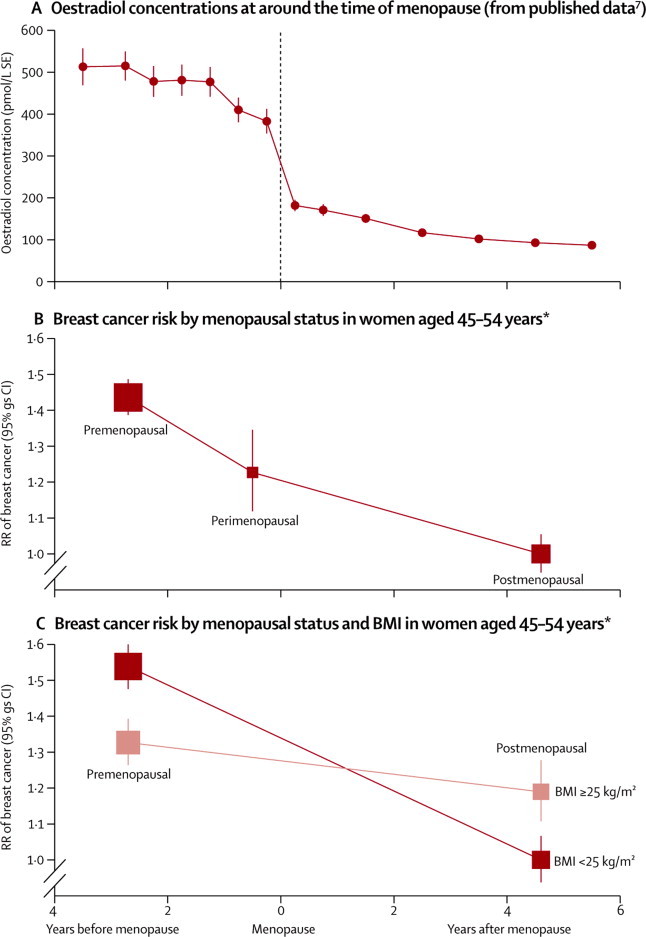
Hormone concentrations and breast cancer risk at around the time of the menopause (A) Circulating oestradiol concentrations, in the years before and after menopause, calculated from published data.^7^ (B) RR of breast cancer in women aged 45–54 years, by menopausal status. (C) RR of breast cancer in women aged 45–54 years by menopausal status and current BMI. RRs stratified by study, age at diagnosis in single years, year of birth, parity, age at first birth, smoking, alcohol consumption, height, and current BMI. RR=relative risk. gs=group specific. BMI=body-mass index. *Results for breast cancer risk are plotted against the time between menopause and the diagnosis of breast cancer for postmenopausal women, and against an estimate of that time for premenopausal and perimenopausal women. The postmenopausal women aged 45–54 years in these analyses reported that their menopause had occurred 4·6 years previously, on average. The premenopausal women aged 45–54 years in these analyses would be expected to reach their menopause in the next 2·7 years, on average (this estimate is based on the age distribution of the premenopausal women in these analyses and the age distribution of women's ages at menopause shown in figure 1B). Perimenopausal women might be expected to reach their menopause in the next 6 months, on average.

Ovarian production of hormones decreases rapidly at around the time of menopause, as is shown in figure 5A, using published data from a cohort study of women who were premenopausal at entry and who had repeated measures of serum oestradiol until after the menopause.^8^ The short-term effect of these changes on breast cancer risk can be assessed by restricting analyses to the 31 000 cases and 70 000 controls aged 45–54 years (since premenopausal, perimenopausal, and postmenopausal women are represented in this age group) and by stratifying analyses by single years of age (so that women of identical ages are being compared). Among such women, breast cancer risk was greater among premenopausal than postmenopausal women (RR 1·43, 95% CI 1·33–1·52, p<0·0001), with the risk for perimenopausal women being between the other two (figure 5B; p<0·001 for all comparisons).

The consistency of the finding of a greater risk of breast cancer among premenopausal than postmenopausal women of the same age was examined across 30 subgroups of women, subdivided by 13 of their characteristics (appendix p 11). There was little heterogeneity across most subgroups, except that women's adiposity attenuated the association. This finding reflects, at least in part, known differences between premenopausal and postmenopausal women in the relation between their current BMI and breast cancer risk, as shown in figure 5C. Among premenopausal women, those who were overweight or obese (BMI ≥25 kg/m^2^) had a lower risk of breast cancer than leaner women (BMI <25 kg/m^2^), whereas the reverse was observed among postmenopausal women. Hence, the RR of breast cancer after the menopause decreases more rapidly in lean than in overweight or obese women (p<0·0001).

**Figure 6:**
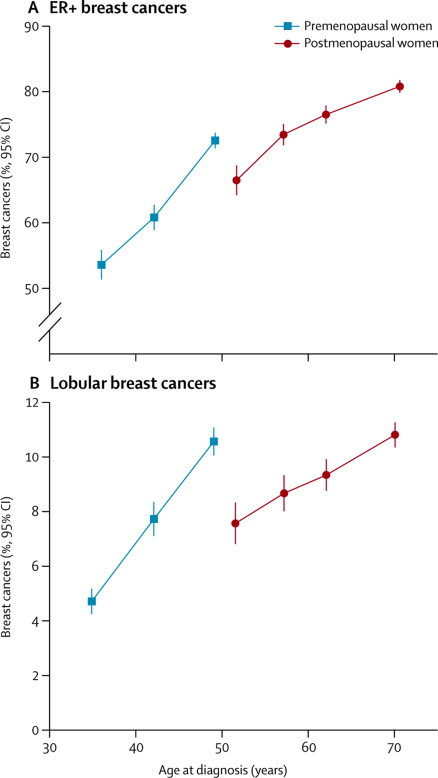
Breast cancer by tumour characteristics and by women's age and menopausal status (A) Oestrogen receptor-positive (ER+). (B) Lobular histology. Results are shown for age groups <40 years, 40–44 years, 45–54 years, 55–59 years, 60–64 years, and ≥65 years, and plotted against the mean age of women with breast cancer in each age group.

The proportion of different types of breast cancer varies by age and by menopausal status (figure 6). For tumours of known oestrogen receptor status, the proportion that is oestrogen receptor-positive increases with age in both premenopausal and postmenopausal women. However, at ages 45–54 years, where both premenopausal and postmenopausal women are represented, a sudden decrease occurs at menopause in the proportion of oestrogen receptor-positive tumours. Similarly, the proportion of tumours with lobular histology increases with age, with a sudden decrease around the time of menopause. After adjusting by single years of age and other potential confounding factors, the heterogeneity at ages 45–54 years between premenopausal and postmenopausal women is highly significant both for oestrogen receptor status (heterogeneity p=0·003) and for tumour histology (heterogeneity p<0·0001, appendix p 11).

In analyses restricted to postmenopausal women, the RR of breast cancer increased by a factor of 1·029 (1·025–1·032, p<0·0001) for every year older at menopause (figure 3B). The findings in figure 3B were stratified by study, year of birth, age, parity and age at first birth, height, current BMI, smoking, and alcohol consumption. Additional adjustment by ethnic origin, age at menarche, family history of breast cancer, and hormonal contraceptive use (both individually and simultaneously) altered the excess RR estimate by less than 1% (data not shown). RRs did not differ significantly between women with a natural menopause (1·030, 1·026–1·034) and bilateral oophorectomy (1·019, 1·004–1·034, heterogeneity p=0·2; appendix p 12).

The association between age at menopause and breast cancer risk was examined in 30 subgroups and did not vary significantly across 11 of the 13 characteristics examined (figure 4B). The association was attenuated by women's current BMI (heterogeneity p=0·008) and by age at first birth (heterogeneity p=0·03). The relation between breast cancer risk and increasing age at menopause was significantly greater for oestrogen receptor-positive disease than for oestrogen receptor-negative disease (p=0·01), and for lobular than for ductal tumours (heterogeneity p=0·006). Cross-classification by both oestrogen receptor status and tumour histology did not show any further interaction (appendix p 6). The findings were not dominated by the results in any particular study (appendix pp 13–15) and there was no significant variation by study design (appendix p 6).

Among postmenopausal women, for whom the effect on breast cancer risk per year younger at menarche can be directly compared to the effect per year older at menopause, breast cancer risk increased by a significantly greater amount per year of menarche than per year of menopause (1·045 [1·036–1·054] *vs* 1·029 [1·025–1·032]; heterogeneity p=0·001).

## Discussion

This worldwide collaboration has brought together and reanalysed individual participant data for 120 000 women with breast cancer and 300 000 controls without the disease from 117 epidemiological studies in 35 countries (panel).

Panel **Research in context**

**Systematic review**

The Collaborative Group on Hormonal Factors in Breast Cancer began in 1992. Since then published literature on epidemiological studies of breast cancer has been identified using electronic searches (Medline, Embase, and PubMed, 1998–2011; using combinations of the search terms “breast cancer”, “risk”, “epidem*”, and “hormones”), supplemented by hand searching in review articles. Eligible studies needed to have obtained individual information about reproductive factors and about use of hormonal therapies, from at least 400 women with breast cancer and similar information from controls without the disease. Studies that had obtained relevant data, but had not published any results for breast cancer were sought by correspondence with colleagues, by discussions at collaborators meetings, and by electronic searches using additional terms “cohort”, “prospective”, “women”, and “cancer risk”.117 eligible studies were included and principal investigators contributed information about almost 120 000 women with breast cancer who had never used menopausal hormonal therapies to this individual-participant meta-analysis. We report on the relation between menarche and menopause and breast cancer risk, overall, and by oestrogen receptor status and by histological subtypes of the tumours, adjusting for possible confounding factors. Subgroup results are also shown by various sociodemographic and personal characteristics, including year of birth, ethnic origin, parity, age at first birth, smoking, alcohol consumption, and body-mass index.

**Interpretation**

Breast cancer risk increased by a significantly greater factor for every year younger at menarche than for every year older at menopause, indicating that menarche and menopause may not affect breast cancer risk merely by extending women's total reproductive years. Endogenous ovarian hormones are more relevant for oestrogen receptor-positive disease than for oestrogen receptor-negative disease and for lobular than for ductal tumours.

While confirming that early menarche and late menopause increase breast cancer risk, we showed that these effects were not equivalent, in that the excess risk associated with lengthening women's reproductive years by one year at menarche was greater than the excess associated with one year's lengthening at menopause. We also found that oestrogen receptor-positive and lobular breast cancers are strongly affected by women's menopausal status, and by their age.

The production of steroid hormones by the ovary begins at around the time of menarche and decreases rapidly at around the time of menopause. Most women become menopausal between the ages of 45 and 54 years. By restricting analyses to the 31 000 women with breast cancer in this narrow age range and stratifying by single years of age (and by other potential confounding factors), valid comparisons can be made of the short-term effect of the menopause, and breast cancer risk was about 40% higher in premenopausal than in postmenopausal women of the same age. Since the postmenopausal women had reached their menopause only an average 4·6 years previously, the findings indicate a rapid decline in breast cancer risk in women of identical ages soon after menopause. This finding probably explains the flattening of the age-incidence curve at around age 50 years, the so-called Clemmesen's hook,^9^ frequently observed in populations before the widespread use of hormonal therapies and screening.

There is accumulating evidence that oestrogen receptor-positive and lobular breast cancers are more sensitive to ovarian hormones than are oestrogen receptor-negative and ductal cancers. Not only are oestrogen receptor-positive and lobular tumours strongly affected by the menopause, as we have shown, but postmenopausal women who use hormone therapy have a greater increase in oestrogen receptor-positive than oestrogen receptor-negative tumours and in lobular than ductal breast cancers.^10^, ^11^ Furthermore, oestrogen-blocking treatments improve survival for oestrogen receptor-positive, but not for oestrogen receptor-negative breast cancer.^12^

Women's adiposity was consistently shown to attenuate associations between menopause and breast cancer risk. Circulating oestradiol concentrations increase as postmenopausal women's BMI increases.^13^ That the RR of breast cancer risk falls more rapidly after the menopause in lean than in overweight and obese women is likely to reflect, at least in part, differences in oestradiol concentrations between such women. Oestradiol concentrations in postmenopausal women are greater the younger they were at menarche,^14^ and this might, in part, account for the associations recorded between age at menarche and breast cancer risk.

Although a woman's age at menarche does not coincide precisely with the onset of breast development, the two are highly correlated.^15^ Breast cancer is almost unknown before menarche and extremely rare soon afterwards, making it effectively impossible to study the short-term effects of the hormonal changes associated with menarche by comparing breast cancer risk in women of identical ages before and soon after menarche.

These findings confirm that young age at menarche and old age at menopause increase breast cancer risk. Many factors known to affect breast cancer risk, including childbearing patterns, height, and BMI, are also associated both with women's age at menarche and with their age at menopause. To ensure as much comparability as possible between women with breast cancer and controls, and thus to minimise potential confounding, analyses were stratified by these factors, and by study, age, year of birth, smoking, and alcohol consumption. Additional adjustment by ethnic origin, hormonal contraceptive use, and family history of breast cancer altered the excess RR estimates by less than 1%. The associations did not vary materially by women's personal characteristics, other than BMI among postmenopausal women.

This meta-analysis of individual-participant data includes almost all available epidemiological evidence for the association between menarche and menopause and breast cancer risk. Large numbers of cases were required to assess reliably whether the associations differ by tumour type and by characteristics of the affected women. Combining individual data from many studies has the advantage of increasing statistical power and also of ensuring that definitions across studies are as similar as possible. Single studies do not have sufficient power to examine these associations in detail, so reviews based solely on the limited published evidence could well be susceptible to publication bias. Although availability of information about oestrogen receptor status varied substantially over time, with most studies done before 1990 contributing relatively little information, analyses of all available data combined were sufficiently powered to describe associations separately within both oestrogen receptor-positive and oestrogen receptor-negative disease. Another important advantage of this meta-analysis is that it seeks to review both published and unpublished findings, thus avoiding unduly selective emphasis on published results or on only some studies.

Women included in these analyses generally reported their ages at menarche and at menopause many years after the events had occurred. Comparisons of information recorded at around the time of menarche and menopause with that recalled many years later has shown substantial regression to the mean over time, especially for recalled age at menarche,^16^, ^17^, ^18^, ^19^, ^20^, ^21^, ^22^, ^23^ which would dilute estimates of RR. 23, 24 The slight attenuation in the estimated RR with attained age thus relects, at least in part, misclassification of recalled ages at menarche and, to a lesser extent, of recalled age at menopause. There might also be systematic differences between cases and controls in reporting their ages at menopause and menarche. Unfortunately insufficient information is available to correct reliably for this misclassification. Since significant associations were recorded at all ages, it is reasonable to conclude that the effects of age at menarche and age at menopause on breast cancer risk persist throughout life.

Since the effect on breast cancer risk of 1 year younger at menarche is significantly greater than that of 1 year older at menopause, these findings suggest that the effects of each may not be acting merely by lengthening the total duration of women's reproductive years. In most populations women's average age at menarche has been declining in successive birth cohorts,^25^ contributing to increasing incidence of breast cancer worldwide.
