# Supplementary material for: Menarche, menopause, and breast cancer risk: individual participant meta-analysis, including 118 964 women with breast cancer from 117 epidemiological studies
Source: Lancet Oncol. 2012 Nov;13(11):1141–51. doi: 10.1016/S1470-2045(12)70425-4 (PMC3488186; doi:10.1016/S1470-2045(12)70425-4)
Supplement: Supplementary appendix [file mmc1.pdf]

THE LANCET Oncology

Supplementary appendix

This appendix formed part of the original submission and has been peer reviewed. We post it as supplied by the authors.

Supplement to: Collaborative Group on Hormonal Factors in Breast Cancer. Menarche, menopause, and breast cancer risk: individual participant meta-analysis, including 118 964 women with breast cancer from 117 epidemiological studies. *Lancet Oncol* 2012; published online Oct 17. http://dx.doi.org/10.1016/S1470-2045(12)70425-4.

Menarche, menopause and breast cancer risk:
individual participant meta-analysis, including 118,964 women with breast cancer from 117 epidemiological studies

Collaborative Group on Hormonal Factors in Breast Cancer

Web Appendix

Correspondence to: Secretariat
Cancer Epidemiology Unit
Richard Doll Building
Oxford OX3 7LF, UK
Tel: +44-(0)-1865-289-600
e-mail: collaborations@ceu.ox.ac.uk

Table of Contents

1. Studies and data: Search strategy, eligibility, data sought, and data checking (p3)

2. Webtable 1. Characteristics of the studies and women included in the analyses (p4-5)

3. Webtable 2. Results by study design (p6)

4. Webtable 3. Subgroup analyses, cross-tabulated by ER staus and histology (p6)

5. Webfigure 1. Associations, only among controls, of various factors with late menarche and early menopause (p7)

6. Webfigure 2. Study-specific results for relative risk of breast cancer per year younger at menarche (p8-10)

7. Webfigure 3. Relative risk of breast cancer in pre-menopausal versus postmenopausal women aged 45-54 years (p11)

8. Webfigure 4. Relative risk of breast cancer by age at natural menopause and age at bilateral oophorectomy (p12)

9. Webfigure 5. Study-specific results for the relative risk of breast cancer per year older at menopause (p13-15)

10. **References** to studies included in these analyses (p16-22)

Search strategy and eligibility of studies for the collaboration

This collaboration began in 1992, and since then potentially eligible epidemiological studies have been sought regularly, by searches of review articles, from computer-aided literature searches in MEDLINE, EMBASE and Pubmed, using combinations of the search terms ‘breast cancer’, ‘cohort’, ‘prospective’, ‘case-control’, ‘hormonal contraceptives’, ‘hormone replacement’, ‘menarche’, ‘menopause’, and ‘reproduct*’. Studies that had collected relevant data, but had not published on breast cancer were sought by correspondence with colleagues, by discussions at collaborators meetings (in 1993, 1995, 2000, 2005 and 2011), and by electronic searches using additional terms ‘cohort’, ‘prospective’, ‘women’ and ‘cancer risk’. To be eligible for the collaboration, epidemiological studies need to have studied at least 400 women with breast cancer (or at least 100 before 2005) and to have collected individual data on women’s childbearing history, and use of hormonal therapies.

Data sought from principal investigators, data checking and collation

Principal investigators were asked to provide individual data from cases and controls on age at menarche, menopausal status, type of and age at menopause (where appropriate), reproductive history, history of hysterectomy and/or bilateral oophorectomy, use of oral contraceptives, use of hormonal therapies for the menopause, height, current weight, weight as a young adult, consumption of alcohol, smoking history, and history of breast cancer in first degree relatives. Principal investigators were also asked to provide information, if available, on tumour histology, estrogen receptor status and a measure of tumour stage.

Data contributed by principal investigators were checked and collated centrally so that analyses could use as similar definitions as possible across studies. Apparent inconsistencies in the data were rectified, where possible, by correspondence with the investigators. After the records had been checked and corrected, investigators were sent summary tables and listings of the variables to be used in analyses for final confirmation that their data had been correctly interpreted.

Studies included in these analyses

Studies were included in these analyses if individual data had been provided on women’s menopausal status, age at menarche and at menopause, and whether or not they had had a hysterectomy or a bilateral oophorectomy. 117 studies had collected relevant data, and they included 44,557 women with breast cancer (38% of the cases) from cohort studies, 50,280 (42%) from case-control studies with population controls and 24,127 (20%) from case-control studies with hospital controls.

Studies included in these analyses and details of the women and breast cancers included in each study are given in Webtable 1 and references to each study are given on p9-13.

Webtable 1. Characteristics of the studies and women, and the breast cancers included in the analyses.

Study (country) ref	Cases/Controls	Cases at diagnosis		Mean age at menarche Cases/Controls	Mean age at natural menopause Cases/Controls	Information on tumour type
		Mean age	Median year			
**35 Cohort studies**						
Thomas (USA) 3	19/74	49.8	1970	12.5/13.2	†	Yes
Kaiser Permanente (USA) 8	22/16	51.2	1974	13.0/12.4	46.3/45.9	No
Adventist Health Study-1 (USA) 23	47/232	67.0	1979	13.3/13.1	49.6/48.5	Yes
Alexander et al (UK) 14	160/628	55.2	1981	13.4/13.5	48.3/48.0	No
Persson (Sweden) 22	26/57	61.8	1982	13.2/13.6	48.8/49.0	No
Canada BC Screening (Canada) 36	779/2,880	51.1	1985	12.9/12.9	49.0/47.8	No
RERF (Japan) 67	179/774	60.9	1985	14.6/15.0	49.7/48.8	No
Guernsey 3&4 (UK) 61	84/286	54.0	1986	13.2/13.1	49.6/49.5	No
BCDDP (USA) 46	845/6,079	63.5	1988	12.8/12.9	49.8/49.0	Yes
Icelandic Study (Iceland) 80	897/8,467	56.6	1989	13.4/13.5	48.3/47.8	No
Nurses Health Study I (USA) 113	3471/14,331	57.7	1990	12.5/12.6	50.1/50.0	Yes
DOM (Netherlands) 70	317/1,011	55.1	1992	13.3/13.4	45.3/44.7	Yes
Iowa (USA) 74	798/3624	68.5	1992	12.8/12.9	49.8/49.6	Yes
NLCS (Netherlands)50	1281/1560	67.4	1992	13.5/13.7	49.6/49.2	Yes
US Radiologic Techno (USA) 93	529/2333	47.7	1992	12.6/12.6	†	No
Petrakis (USA) 12	204/747	59.0	1993	12.7/12.8	46.1/46.8	No
Amer.Can.Soc. (USA) 89‡	3126/12,236	67.2	1994	12.7/12.9	49.5/49.0	No
Newcomb/Mandelson (USA) 55	34/63	44.5	1994	12.1/12.1	†	Yes
SOF (USA) 98	162/665	76.7	1994	13.1/13.1	48.6/47.9	Yes
Swedish Mammography (Sweden) 102	1125/3164	63.9	1994	13.2/13.4	50.1/49.7	Yes
Shanghai Textile (China) 88	1376/5458	54.4	1995	15.3/15.5	49.4/48.9	Yes
ACS Nutrition (USA) 82	1141/5692	67.3	1997	12.7/12.8	49.8/49.5	Yes
Danish Nurses (Denmark) 96	99/550	64.3	1997	13.7/13.9	50.8/49.5	Yes
Miyagi (Japan) 112	185/719	55.7	1997	14.4/14.7	50.6/49.8	Yes
NIH-AARP (USA) 106	3352/14,191	66.1	1999	12.0/12.1	48.8/48.3	Yes
JPHC (Japan) 116	569/2209	59.4	2000	14.4/14.6	49.8/49.4	Yes
Multi Ethnic Cohort (USA) 99	1785/14,360	64.9	2000	12.6/12.7	48.7/48.3	Yes
Nurses Health Study II (USA) 113	1774/6099	45.4	2000	12.3/12.4	†	Yes
WHI (USA) 86	628/2958	69.6	2000	12.6/12.7	50.7/50.2	Yes
Southern Sweden (Sweden) 92	752/3135	57.5	2001	13.3/13.4	47.9/48.2	Yes
S/N Womens (Norway/Sweden) 94	706/3044	50.2	2001	13.0/13.0	†	Yes
EPIC (10 European Countries) 108	6474/27,388	57.3	2002	13.0/13.1	49.8/49.2	Yes
Million Women Study (UK) 114	8724/38,068	59.3	2002	12.9/13.0	50.0/49.4	Yes
NOWAC (Norway) 90	403/2,044	63.5	2002	13.3/13.5	49.3/49.2	Yes
UKCTOCS (UK) 117	2484/7687	64.8	2007	12.9/13.0	50.7/50.1	Yes
**Subtotal**	**44,557/192,829**	**60.2**	**1999**	**12.9/13.0**	**49.8/49.4**	
**56 Case control studies (with population controls)**						
Leisure World (USA) 2	24/60	69.0	1974	13.2/13.3	46.3/46.7	Yes
Brinton (USA) 5	1,759/1,910	52.6	1976	12.8/12.9	48.2/47.7	No
Ursin (USA) 38	112/152	39.5	1977	12.5/12.6	†	No
Nomura (USA) 9	151/129	57.2	1978	13.2/13.3	48.9/48.9	Yes
Bernstein/Pike (USA) 1	430/418	31.5	1980	12.3/12.5	†	Yes
CASH (USA) 31	3,174/3,138	43.0	1981	12.6/12.7	†	Yes
Hislop (Canada) 7	565/591	50.7	1981	13.1/13.1	49.2/49.2	Yes
Bain (Australia) 20	373/748	54.3	1983	13.1/13.2	48.5/48.2	Yes
Ewertz (Denmark) 15	964/918	49.9	1983	13.6/13.7	†	Yes
Oberle (Costa Rica) 11	154/781	44.8	1983	13.4/13.4	†	No
Rohan (Australia) 17	307/324	53.5	1983	13.2/13.2	49.1/48.1	Yes
UK National (UK) 21	743/744	31.9	1983	12.6/12.7	†	Yes
Clarke (Canada) 37	456/942	50.1	1984	12.8/12.9	49.6/48.5	Yes
Long Island Study (USA) 30	880/907	54.5	1984	12.7/12.7	48.8/49.2	Yes
Meirik/Lund (Sweden/Norway) 24	386/513	37.2	1984	13.0/13.0	†	No
Yuan/Yu (China) 19	530/531	50.7	1984	14.6/15.0	48.0/47.5	Yes
American Asian (USA) 59	443/848	44.4	1985	12.9/13.1	†	Yes
Wrensch/Baltzell (USA) 81	459/2,348	50.4	1985	12.5/12.6	48.0/46.2	No
Paul/Skegg (New Zealand) 63	699/1,573	44.2	1985	12.9/13.0	†	No
Wang/Yu (China) 39	294/294	43.2	1985	14.1/14.4	†	Yes
Bernstein II (USA) 64	676/675	35.5	1986	12.3/12.5	†	No
Daling (USA) 47	659/859	36.5	1987	12.6/12.7	†	Yes
Western New York (USA) 34	547/591	53.6	1987	12.6/12.9	48.8/47.9	Yes
CRC/ICRF (UK) 97	570/564	41.5	1988	12.6/12.7	†	Yes

Dutch OC (Netherlands) 56	823/805	41.6	1988	12.9/13.0	†	Yes
Newcomb (USA) 75	4,600/6,793	56.6	1988	12.9/13.0	49.0/48.3	No
Sanjose (Spain) 53	285/308	55.8	1988	13.1/12.9	49.3/48.3	No
Ross/Pike (USA) 76	799/788	61.2	1989	12.6/12.7	49.9/49.8	Yes
Stanford/Habel (USA) 57	184/205	57.5	1989	12.6/12.5	49.5/49.4	Yes
Yang/Gallagher (Canada) 40	525/523	53.8	1989	12.5/12.6	48.7/48.3	Yes
Zakelj (Slovenia) 49	612/611	44.8	1989	13.8/13.9	†	Yes
ICRF (UK) 104	277/277	49.5	1991	12.9/12.9	†	Yes
WISH (USA) 60	1,635/1,690	40.1	1991	12.4/12.5	†	Yes
4-State Wisconsin (USA) 72	2,813/2,920	65.7	1992	12.9/12.9	49.6/48.9	Yes
McCredie (Australia) 62	431/394	34.8	1993	12.9/12.9	†	Yes
Morabia II (Switzerland) 54	134/665	53.4	1993	13.0/13.4	48.8/48.1	No
ChangClaude (Germany) 87	559/1115	41.8	1994	13.1/13.1	†	Yes
Magnusson (Sweden) 68	1572/1973	62.6	1994	13.6/13.7	50.6/50.1	Yes
Millikan (USA) 69	441/375	41.7	1994	12.5/12.5	†	No
Johnson (Canada) 73	1652/1663	54.5	1995	12.8/12.9	49.0/48.4	Yes
CARE (USA) 85	2611/2546	46.1	1996	12.4/12.4	†	Yes
Freindenreich (Canada) 78	699/667	52.7	1996	12.7/12.7	51.1/51.0	Yes
McCredie II (Australia) 65	753/429	41.3	1996	12.8/12.8	†	Yes
Kreiger (Canada) 83	1816/1868	53.7	1997	12.8/12.9	49.0/48.8	Yes
LIBCSP (USA) 105	830/998	56.6	1997	12.4/12.4	48.9/48.6	Yes
Lumachi (Italy) 84	272/312	55.0	1997	12.3/12.8	50.8/48.9	Yes
Newcomb3 (USA) 77	3586/4493	50.7	1997	12.7/12.8	49.5/48.9	Yes
San Francisco Bay (USA) 91	1189/1473	52.2	1997	12.6/12.8	47.4/47.0	Yes
Shu Wei (China) 79	1397/1481	47.6	1997	14.5/14.7	†	No
PACE (USA) 100	210/265	72.5	1998	12.9/13.0	49.2/49.0	Yes
WISE (USA) 103	449/711	63.8	2000	12.6/12.8	48.9/48.7	Yes
Polish Breast Cancer (Poland) 101	1,434/1907	56.2	2001	13.5/13.7	49.9/49.5	Yes
Zhou Xin (China) 95	204/409	46.2	2002	13.2/15.0	†	Yes
MARIE (Germany) 107	855/2185	61.8	2003	13.7/13.7	49.8/49.4	Yes
MASTOS (Cyprus) 110	583/832	53.4	2003	13.0/13.2	50.6/50.0	No
CAMA (Mexico) 109	695/851	50.5	2006	12.8/12.9	47.1/47.0	Yes
**Subtotal**	**50,280/ 63,090**	**50.8**	**1992**	**12.9/13.0**	**49.0/48.5**	
**26 Case control studies (with hospital controls)**						
Morabia I(USA) 41	299/404	48.6	1974	12.8/13.0	†	Yes
Vessey I (UK) 4	1134/1044	40.9	1976	12.9/13.2	†	Yes
Modan (Israel) 10	925/1718	55.0	1977	13.2/13.2	49.3/48.7	No
Hulka I (USA) 26	154/1285	54.2	1978	13.0/13.0	47.1/46.5	No
Franceschi I (Italy) 29	279/321	57.7	1980	13.4/13.7	48.3/48.0	No
Kalache (Brazil) 43	532/730	48.4	1980	13.6/13.6	47.3/47.1	No
Ravnihar (Slovenia) 16	493/1,747	44.2	1981	14.0/14.2	†	Yes
Vessey II (UK) 13	923/844	47.5	1982	13.1/13.1	†	Yes
WHO - developed 42	1,375/3,578	43.2	1982	13.2/13.2	†	Yes
- developing 42	1,519/14,669	42.8	1984	14.6/14.7	†	Yes
Lê (France) 6	257/249	38.1	1983	12.6/12.9	†	No
CCSS (USA) 28	4564/5097	48.6	1984	12.6/12.7	†	Yes
Clavel (France) 27	438/791	43.5	1984	12.9/13.2	†	Yes
Gerber (France) 32	328/427	51.0	1984	13.0/13.1	50.7/48.9	Yes
Marubini (Italy) 18	166/175	48.0	1984	13.0/12.7	†	Yes
La Vecchia (Italy) 51	2,877/2,401	52.3	1986	12.9/13.0	49.5/48.5	No
Lee (Singapore) 35	196/404	50.6	1987	13.6/14.1	49.5/48.4	Yes
PEDS (USA) 25	621/2,287	55.0	1987	12.7/12.8	49.6/49.1	Yes
Ngelangel (Philippines) 42	271/279	46.1	1988	13.7/13.7	†	Yes
Hulka II (USA) 45	65/125	42.2	1990	12.9/12.6	†	Yes
Katsouyanni (Greece) 48	656/1,263	54.9	1990	12.9/13.1	48.6/47.8	Yes
Franceschi II (Italy) 58	2,243/2,206	53.9	1992	12.9/13.0	49.9/49.1	No
Hamajima (Japan) 66	1338/5441	50.5	1992	13.8/14.0	49.9/49.4	No
Levi (Switzerland) 52	164/375	53.6	1992	13.4/13.5	49.7/49.9	Yes
Gajalakshmi (India) 33	466/485	48.4	1993	13.9/14.0	†	No
Johannesburg (South Africa) 71	1424/1,367	51.0	2003	15.3/15.3	48.2/47.8	Yes
Ghiasvand (Iran) 115	420/460	40.3	2006	13.9/14.0	†	No
**Subtotal**	**24,127/50,172**	**49.1**	**1985**	**13.3/13.7**	**48.9/47.9**	
**TOTAL**	**118,964/306,091**	**54.0**	**1993**	**13.0/13.1**	**50.0/49.5**	

† The average age at cancer diagnosis was <50 years, and so the average age at menopause of the few postmenopausal women is uninterpretable, as it reflects the age-restriction of cases and controls. However, all women are included in the analyses.
‡ Cases were of fatal breast cancer

Webtable 2. Relative risk of breast cancer by age at menarche and age at menopause, by study design

	Relative risk (95%CI) per year younger at menarche
**Women aged <50 years**	
Cohort studies	1.047 (1.021-1.074)
Case-control studies, population controls	1.066 (1.049-1.083)
Case-control studies, hospital controls	1.076 (1.057-1.097)
	Heterogeneity by study design, p=0.2
**Women aged 50+**	
Cohort studies	1.039 (1.029-1.049)
Case-control studies, population controls	1.051 (1.034-1.069)
Case-control studies, hospital controls	1.034 (1.017-1.065)
	Heterogeneity by study design, p=0.5
	**Relative risk (95%CI) per year older at menopause**
Cohort studies	1.030 (1.025-1.034)
Case-control studies, population controls	1.024 (1.017-1.031)
Case-control studies, hospital controls	1.032 (1.023-1.043)
	Heterogeneity by study design, p=0.3

*Relative risk estimates are calculated stratifying, by study, age, year of birth, parity, age at first birth, smoking, alcohol consumption, height, and current body mass index.

Webtable 3. Relative risk of breast cancer by age menarche and age at menopause, by ER status and tumour histology

	Relative risk (95%CI) per year younger at menarche
Ductal cancer, ER+ve	1.034 (1.026-1.052)
Ductal cancer, ER-ve	1.024 (1.004-1.044)
Lobular cancer, ER+ve	1.083 (1.052-1.115)
Lobular cancer, ER-ve	1.076 (0.999-1.159)
	**Relative risk (95%CI) per year older at menopause**
Ductal cancer, ER+ve	1.026 (1.020-1.032)
Ductal cancer, ER-ve	1.010 (1.000-1.020)
Lobular cancer, ER+ve	1.046 (1.031-1.061)
Lobular cancer, ER-ve	1.032 (0.994-1.072)

*Relative risk estimates are calculated stratifying, by study, age and year of birth, and adjusting by parity, age at first birth, smoking, alcohol consumption, height, and current body mass index.

Webfigure 1. Associations, only among controls, of various factors with:
(A) menarche at or after age 15 years vs at younger ages and
(B) natural menopause at or before age 45 years vs at older ages

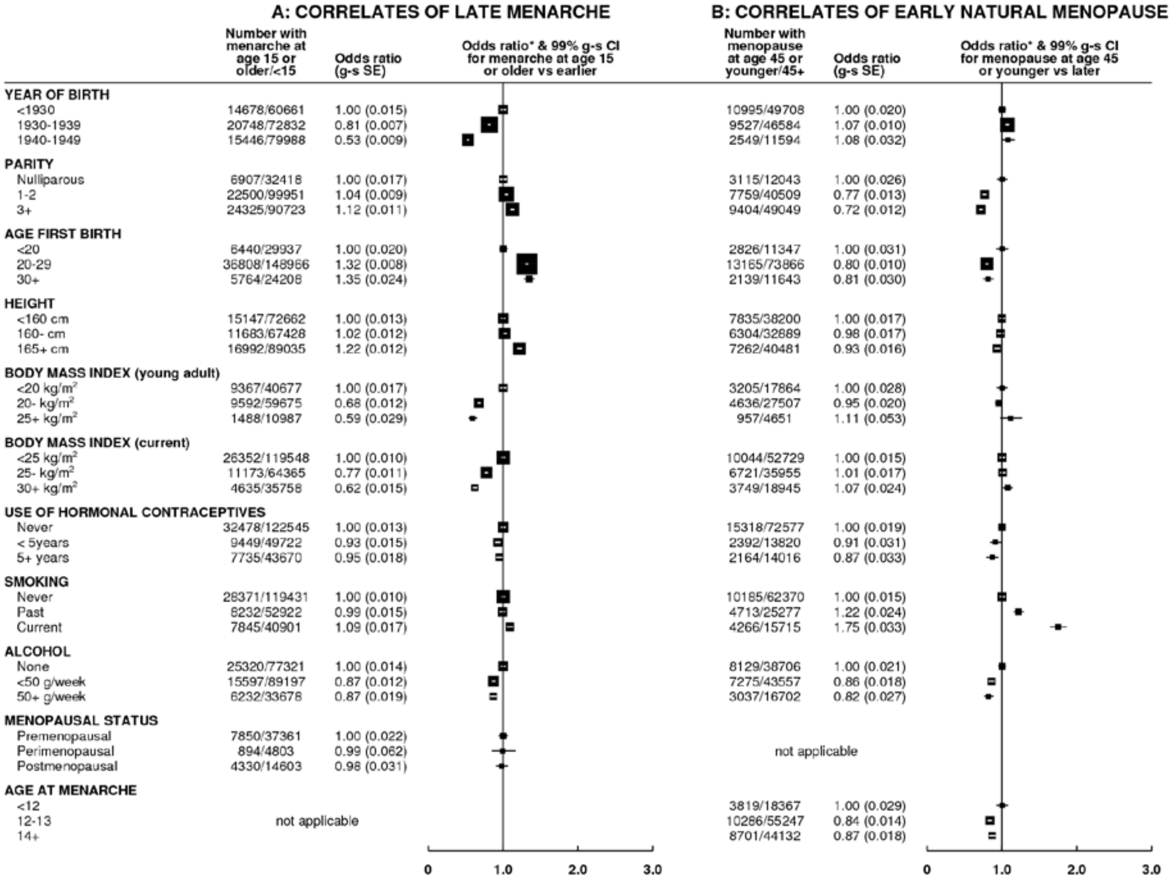

Data are for women without breast cancer, ie controls. Odds ratio calculations were stratified by study and, where appropriate, by age at diagnosis, year of birth, parity, age at first birth, smoking, alcohol consumption, height, and current body mass index (results for body mass index as a young adult are not stratified by current body mass index).

Webfigure 2A. **Relative risk of breast cancer per year younger at menarche in cohort studies.**

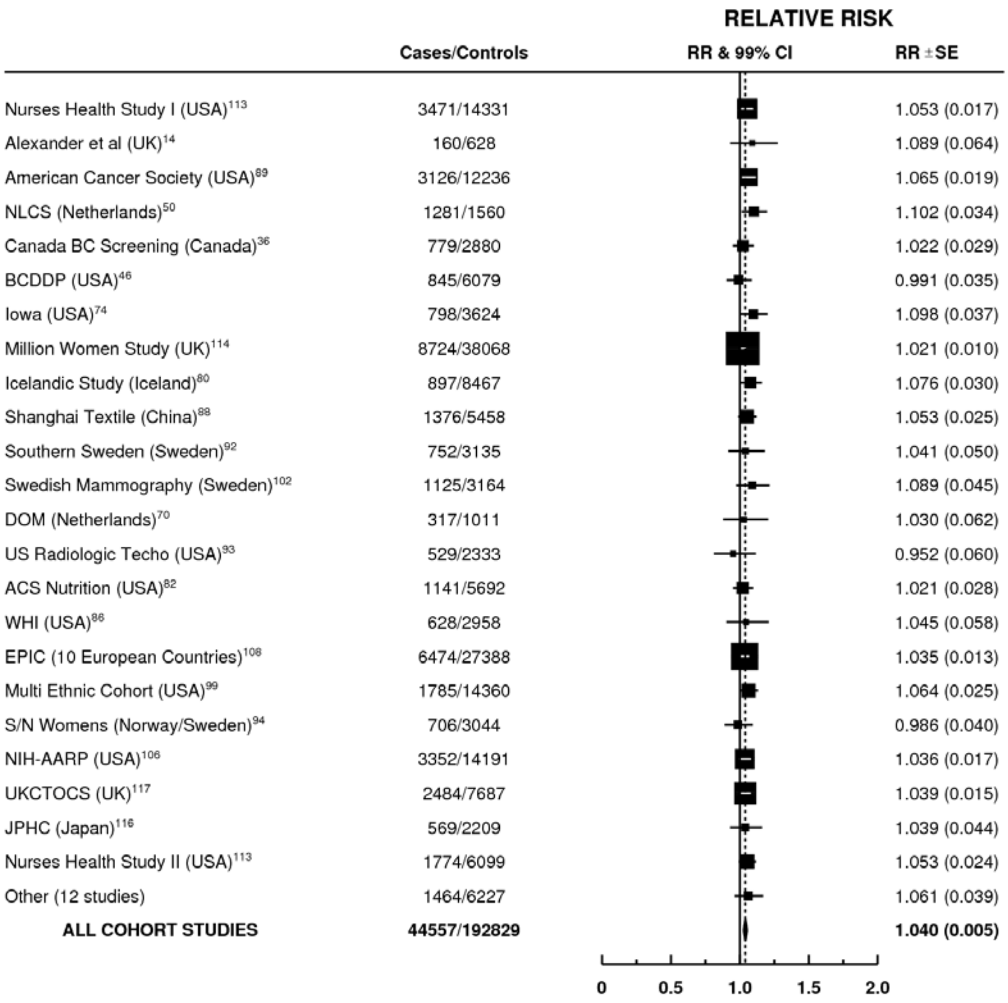

Relative risks are calculated stratifying by age at diagnosis, year of birth, parity, age at first birth, smoking, alcohol consumption, height, and current body mass index.

Webfigure 2B. Relative risk of breast cancer per year younger at menarche in case-control studies with population controls.

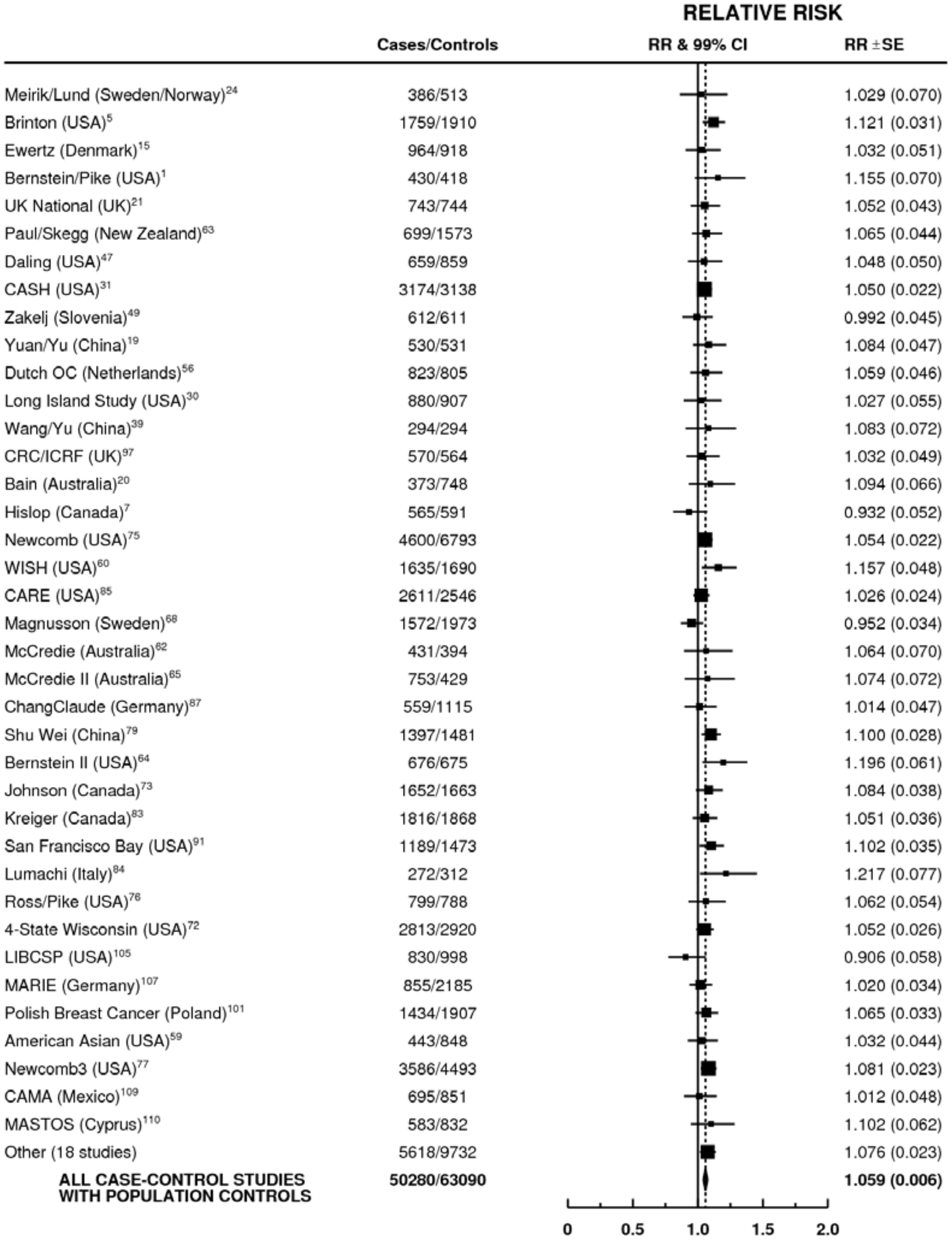

Relative risks are calculated stratifying by age at diagnosis, year of birth, parity, age at first birth, smoking, alcohol consumption, height, and current body mass index.

Webfigure 2C. Relative risk of breast cancer per year younger at menarche in case-control studies with hospital controls.

RELATIVE RISK

	Cases/Controls	RR ±SE
Clavel (France)[27]	438/791	1.081 (0.059)
La Vecchia (Italy)[51]	2877/2401	1.066 (0.025)
Ravnihar (Slovenia)[16]	493/1747	1.066 (0.036)
Vessey I (UK)[4]	1134/1044	1.103 (0.032)
Vessey II (UK)[13]	923/844	1.025 (0.043)
Morabia I(USA)[41]	299/404	1.072 (0.061)
Kalache (Brazil)[43]	532/730	1.053 (0.046)
Franceschi II (Italy)[58]	2243/2206	1.048 (0.027)
Hamajima (Japan)[66]	1338/5441	1.078 (0.025)
Katsouyanni (Greece)[48]	656/1263	1.099 (0.058)
Modan (Israel)[10]	925/1718	1.016 (0.033)
Gajalakshmi (India)[33]	466/485	1.064 (0.058)
PEDS (USA)[25]	621/2287	0.994 (0.064)
Johannesburg (South Africa)[71]	1424/1367	1.069 (0.032)
WHO - developing[42]	1519/14669	1.050 (0.020)
WHO - developed[42]	1375/3578	1.038 (0.023)
CCSS (USA)[28]	4564/5097	1.106 (0.028)
Other (10 studies)	2300/4100	1.086 (0.035)
**ALL CASE-CONTROL STUDIES WITH HOSPITAL CONTROLS**	**24127/50172**	**1.062 (0.008)**

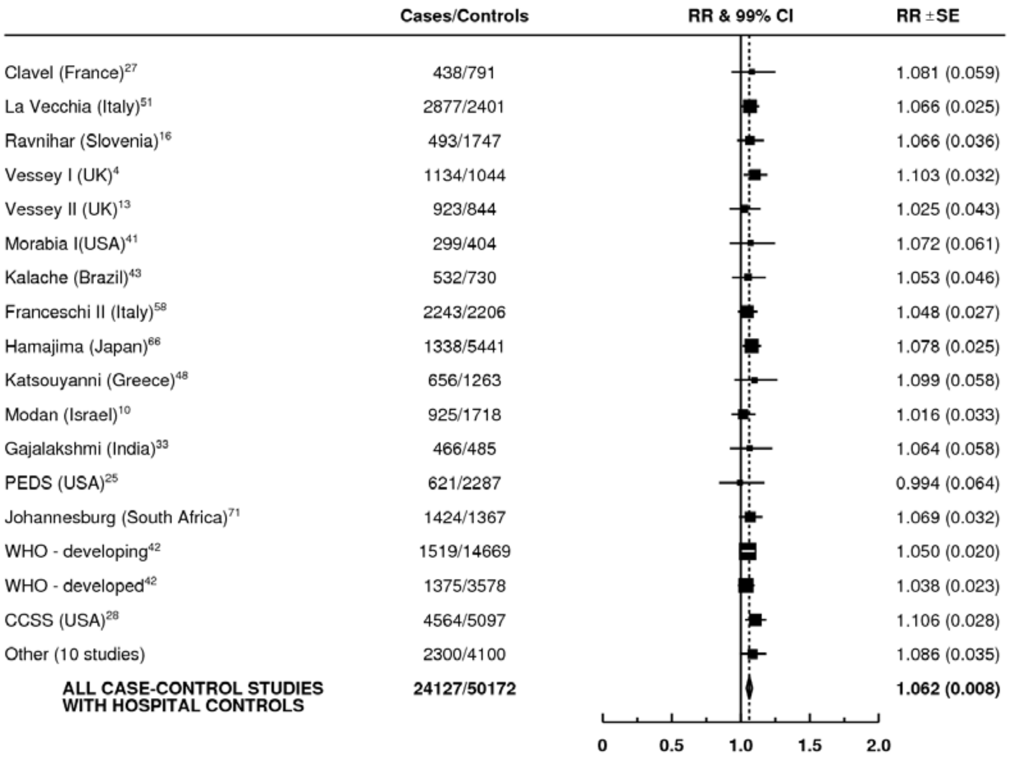

Relative risks are calculated stratifying by age at diagnosis, year of birth, parity, age at first birth, smoking, alcohol consumption, height, and current body mass index.

Webfigure 3. Relative risk of breast cancer in pre-menopausal versus postmenopausal women aged 45- 54 years in subgroups of women;* and by tumour characteristics‡

	Premenopausal Cases/Controls	Postmenopausal Cases/Controls	RR* (SE)
**ALL WOMEN**	**20744/45211**	**8170/18933**	**1.43 (0.040)**
**WOMEN'S CHARACTERISTICS**			
**Year of birth**			
<1940	7014/13566	4411/9526	1.45 (0.056)
1940+	13730/31645	3759/9407	1.38 (0.055)
Heterogeneity test: χ^2_1=0.5, P=0.5			
**Age at diagnosis**			
45-49	12620/24650	1648/3625	1.39 (0.068)
50-54	8124/20561	6522/15308	1.44 (0.050)
Heterogeneity test: χ^2_1=0.3, P=0.6			
**Ethnicity**			
Caucasian	12434/23542	4747/9875	1.38 (0.055)
Non-Caucasian	3685/9506	1879/4696	1.44 (0.081)
Heterogeneity test: χ^2_1=0.3, P=0.6			
**Parity**			
Nulliparous	2691/4894	1230/2147	1.37 (0.125)
Parous	17582/38569	6705/16022	1.41 (0.038)
Heterogeneity test: χ^2_1=0.1, P=0.7			
**Age at first birth**			
<25	8946/22367	3806/10097	1.37 (0.049)
25+	8739/16870	2920/6069	1.47 (0.060)
Heterogeneity test: χ^2_1=1.2, P=0.3			
**Height**			
<165 cm	10670/22895	4503/9721	1.33 (0.051)
165+ cm	8146/16242	2876/6125	1.47 (0.071)
Heterogeneity test: χ^2_1=1.9, P=0.2			
**Body mass index (young adult)**			
<20 kg/m^2	4960/8571	1713/3059	1.39 (0.093)
20- kg/m^2	5925/11226	2027/3933	1.33 (0.080)
25+ kg/m^2	802/1846	354/828	1.57 (0.367)
Heterogeneity test: χ^2_2=0.4, P=0.8			
**Body mass index (current)**			
<25 kg/m^2	11423/22540	3888/8504	1.51 (0.059)
25- kg/m^2	4306/9728	1951/4244	1.17 (0.088)
30+ kg/m^2	2061/5279	1012/2302	1.08 (0.135)
Heterogeneity test: χ^2_2=11.0, P=0.04			
**Use of hormonal contraceptives**			
Never	8247/16081	4766/9956	1.43 (0.063)
Ever	11252/25985	2865/7581	1.50 (0.072)
Heterogeneity test: χ^2_1=0.4, P=0.5			
**Smoking**			
Never	9732/21706	3543/7715	1.32 (0.055)
Past	4370/9357	1407/2946	1.55 (0.120)
Current	3849/7527	1896/3983	1.44 (0.108)
Heterogeneity test: χ^2_2=2.7, P=0.3			
**Alcohol**			
None	5567/13311	2499/6098	1.30 (0.069)
<50 g/week	7200/16754	2121/5249	1.41 (0.085)
50+ g/week	3445/5353	1378/2519	1.71 (0.155)
Heterogeneity test: χ^2_2=4.4, P=0.1			
**Mother or sister with breast cancer**			
No	14958/33288	6255/14734	1.39 (0.046)
Yes	2380/2959	955/1236	1.53 (0.275)
Heterogeneity test: χ^2_1=0.2, P=0.7			
**Age at menarche**			
<13	8385/17122	3229/6482	1.24 (0.082)
13+	12359/28089	4941/12451	1.51 (0.056)
Heterogeneity test: χ^2_1=5.4, P=0.02			
**TUMOUR CHARACTERISTICS**			
**ER status**			
negative	1533/22149	552/7957	1.26 (0.077)
positive	4054/22149	1095/7957	1.53 (0.060)
case-case comparison for ER+ vs. ER-: p=0.003			
**Histology**			
ductal	10160/34440	3572/13374	1.33 (0.033)
lobular	1456/34440	352/13374	2.05 (0.109)
other	2162/34440	726/13374	1.47 (0.069)
case-case comparison for lobular vs. ductal: p<0.0001			

Relative risk* of breast cancer in premenopausal vs postmenopausal & 99% CI

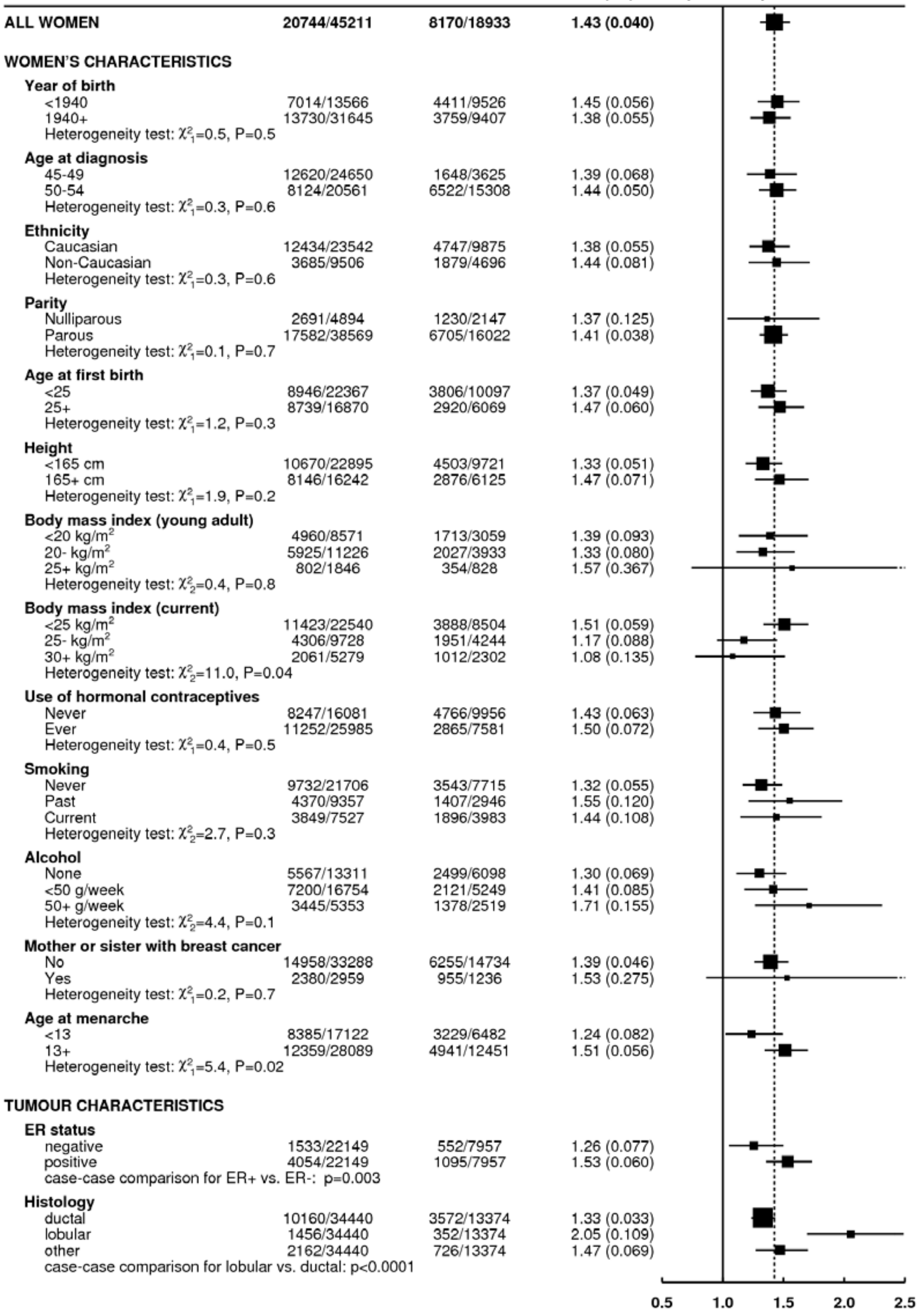

*Relative risks are calculated stratifying by study, age at diagnosis in single years, and, where appropriate, by year of birth, parity, age at first birth, smoking, alcohol consumption, height, and current body mass index (results for body mass index as a young adult are not stratified by current body mass index).

‡Relative risks are calculated stratifying by study, age at diagnosis in single years, and year of birth, and adjusting by parity, age at first birth, smoking, alcohol consumption, height and current body mass index. Case-case comparisons stratified by study, age and year of birth, and adjusted by parity, age at first birth, smoking, alcohol consumption, height , and current body mass index.

Webfigure 4. Relative risk of breast cancer by:
(A) age at natural menopause, and (B) age at bilateral oophorectomy.

A: Age at natural menopause

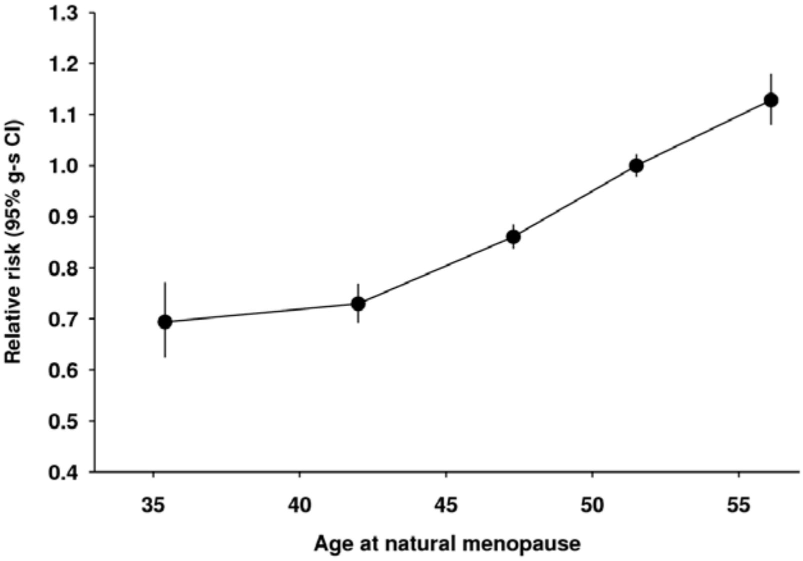

Age group (mean, years)	<40 (35.4)	40-44 (42.0)	45-49 (47.3)	50-54 (51.5)	55+ (56.1)
Cases/Controls	1384/4356	4649/15640	16137/49089	27370/74142	6677/15657
RR (95% g-s CI)	0.69 (0.62-0.77)	0.73 (0.69-0.77)	0.86 (0.84-0.88)	1.00 (0.98-1.02)	1.13 (1.08-1.18)

B: Age at bilateral oophorectomy

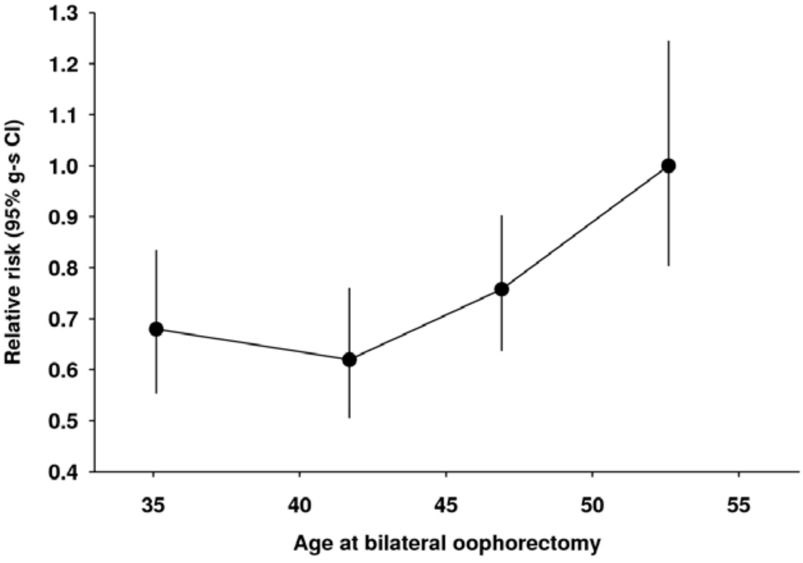

Age group (mean, years)	<40 (35.1)	40-44 (41.7)	45-49 (46.9)	50+ (52.6)
Cases/Controls	970/3267	845/2839	1167/2873	979/2178
RR (95% g-s CI)	0.68 (0.55-0.83)	0.62 (0.51-0.76)	0.76 (0.64-0.90)	1.00 (0.80-1.24)

*Relative risk estimates are calculated stratifying by study, age, year of birth, parity, age at first birth, smoking, alcohol consumption, height and current body mass index.

Relative risks in pre-menopausal versus postmenopausal women aged 45-54 years with a natural menopause and with a bilateral oophorectomy were 1.42 (95%CI 1.32-1.52) and 1.53 (95%CI 1.26-1.86), respectively, (p for heterogeneity=0.6).

Webfigure 5A. Relative risk of breast cancer per year older at menopause in cohort studies.

RELATIVE RISK

	Cases/Controls	RR ±SE
Nurses Health Study I (USA)[113]	2173/9761	1.040 (0.008)
Alexander et al (UK)[14]	111/455	1.004 (0.027)
American Cancer Society (USA)[89]	1701/6652	1.033 (0.010)
NLCS (Netherlands)[50]	1281/1560	1.026 (0.014)
Canada BC Screening (Canada)[36]	248/995	1.066 (0.022)
BCDDP (USA)[46]	845/2117	1.055 (0.018)
RERF (Japan)[67]	128/569	1.102 (0.058)
Iowa (USA)[74]	798/3624	1.012 (0.011)
Million Women Study (UK)[114]	6119/28370	1.028 (0.005)
Icelandic Study (Iceland)[80]	595/5592	1.024 (0.012)
Shanghai Textile (China)[88]	816/3267	1.030 (0.012)
Southern Sweden (Sweden)[92]	332/1548	1.023 (0.036)
Swedish Mammography (Sweden)[102]	955/3145	1.069 (0.015)
NOWAC (Norway)[90]	401/2035	0.991 (0.025)
SOF (USA)[98]	162/665	1.051 (0.033)
ACS Nutrition (USA)[82]	1074/5412	1.016 (0.010)
WHI (USA)[86]	628/2958	1.051 (0.019)
EPIC (10 European Countries)[108]	2266/10905	1.037 (0.009)
Multi Ethnic Cohort (USA)[99]	1209/10295	1.017 (0.010)
S/N Womens (Norway/Sweden)[94]	116/542	0.963 (0.055)
NIH-AARP (USA)[106]	3217/13641	1.025 (0.005)
UKCTOCS (UK)[117]	2484/7687	1.032 (0.006)
JPHC (Japan)[116]	277/1132	1.049 (0.029)
Other	655/2680	1.051 (0.036)
**ALL COHORT STUDIES**	**28591/125607**	**1.030 (0.002)**

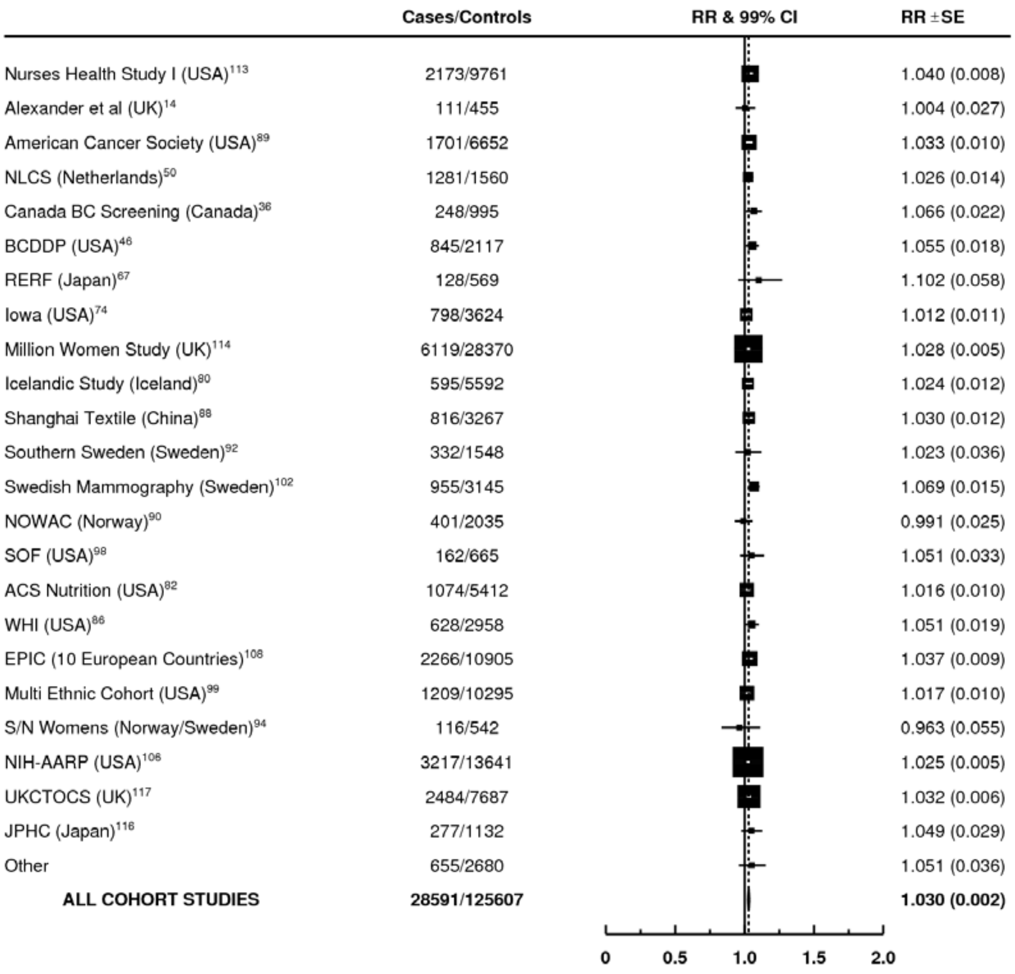

Relative risks are calculated stratifying by age at diagnosis, year of birth, parity, age at first birth, smoking, alcohol consumption, height, and current body mass index.

Webfigure 5B. Relative risk of breast cancer per year older at menopause in case-control studies with population controls.

RELATIVE RISK

	Cases/Controls	RR ±SE
Brinton (USA)[5]	790/947	1.012 (0.015)
Ewertz (Denmark)[15]	388/393	1.061 (0.039)
Rohan (Australia)[17]	180/202	1.062 (0.064)
Paul/Skegg (New Zealand)[63]	105/152	0.978 (0.060)
CASH (USA)[31]	365/456	0.984 (0.033)
Zakelj (Slovenia)[49]	111/142	0.995 (0.065)
Yuan/Yu (China)[19]	297/298	1.051 (0.025)
Long Island Study (USA)[30]	533/575	0.957 (0.026)
Yang/Gallagher (Canada)[40]	313/311	1.038 (0.040)
Stanford/Habel (USA)[57]	149/165	1.011 (0.055)
Bain (Australia)[20]	226/475	1.019 (0.035)
Hislop (Canada)[7]	277/291	1.001 (0.030)
Newcomb (USA)[75]	3029/4108	1.021 (0.009)
Clarke (Canada)[37]	213/437	1.013 (0.047)
CARE (USA)[85]	621/610	1.038 (0.021)
Sanjose (Spain)[53]	186/220	0.950 (0.043)
Magnusson (Sweden)[68]	1433/1867	1.047 (0.015)
ChangClaude (Germany)[87]	66/121	0.955 (0.067)
Shu Wei (China)[79]	457/505	1.038 (0.019)
Johnson (Canada)[73]	845/854	1.045 (0.019)
Friedenreich (Canada)[78]	302/269	1.068 (0.047)
Kreiger (Canada)[83]	877/844	1.005 (0.017)
San Francisco Bay (USA)[91]	502/639	1.025 (0.018)
Lumachi (Italy)[84]	151/174	1.287 (0.073)
PACE (USA)[100]	210/265	1.008 (0.033)
Ross/Pike (USA)[76]	799/787	1.033 (0.020)
4-State Wisconsin (USA)[72]	2574/2660	1.026 (0.008)
LIBCSP (USA)[105]	487/513	1.039 (0.027)
MARIE (Germany)[107]	723/1877	1.022 (0.014)
Polish Breast Cancer (Poland)[101]	974/1266	1.036 (0.017)
Western New York (USA)[34]	258/289	1.039 (0.041)
American Asian (USA)[59]	76/194	1.068 (0.070)
Newcomb3 (USA)[77]	1340/1805	1.028 (0.013)
CAMA (Mexico)[109]	339/396	0.940 (0.024)
MASTOS (Cyprus)[110]	410/475	1.025 (0.028)
WISE (USA)[103]	378/597	0.956 (0.028)
Wrensch/Baltzell (USA)[81]	186/393	1.007 (0.070)
Other	722/1191	1.123 (0.043)
**ALL CASE-CONTROL STUDIES WITH POPULATION CONTROLS**	**21892/27763**	**1.024 (0.003)**

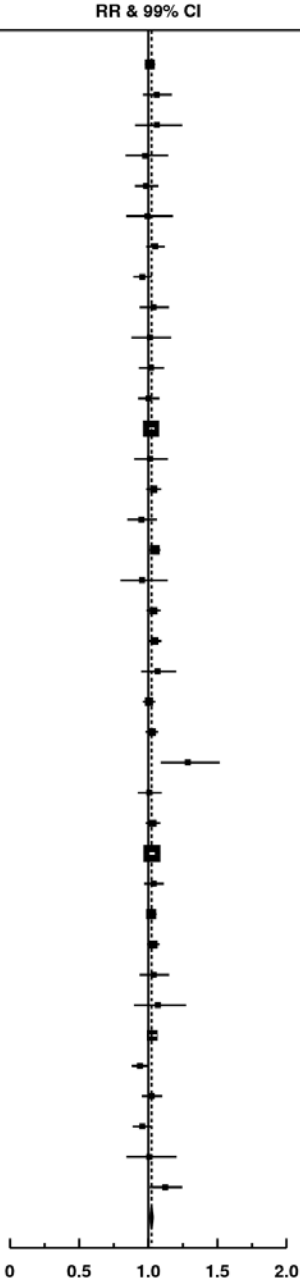

Relative risks are calculated stratifying by age at diagnosis, year of birth, parity, age at first birth, smoking, alcohol consumption, height, and current body mass index.

Webfigure 5C. Relative risk of breast cancer per year older at menopause in case-control studies with hospital controls.

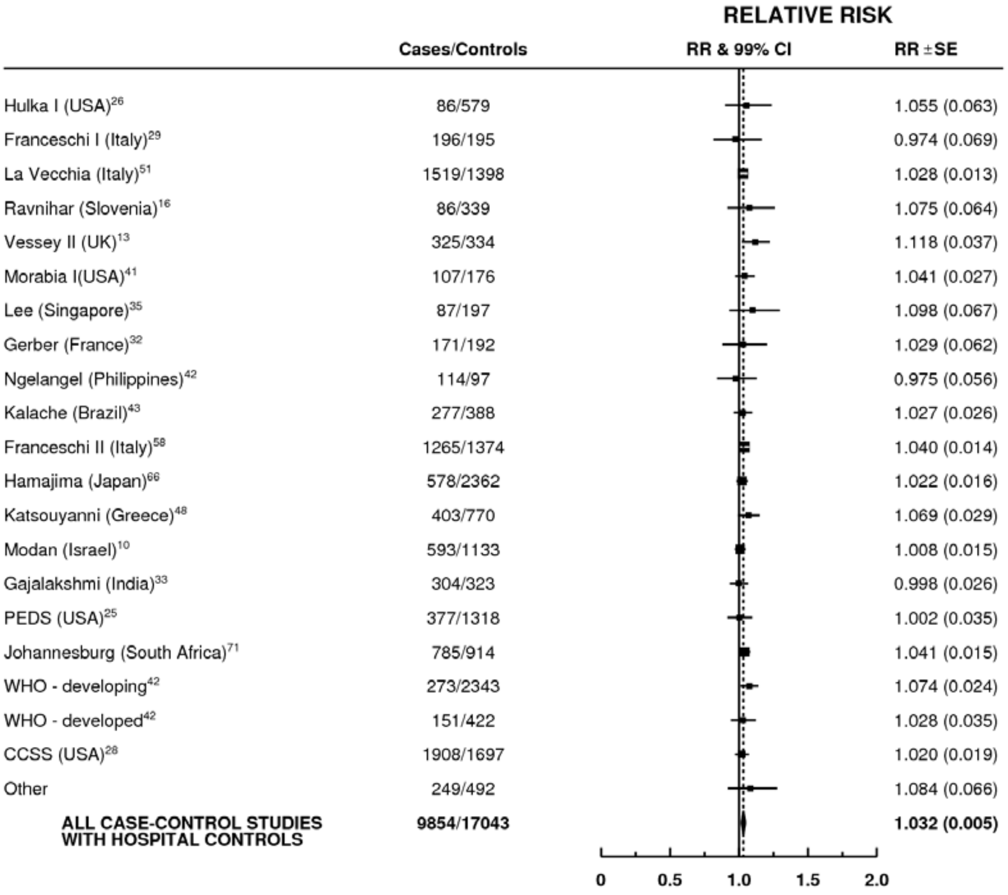

Relative risks are calculated stratifying by age at diagnosis, year of birth, parity, age at first birth, smoking, alcohol consumption, height, and current body mass index.

REFERENCES

1. Pike MC, Henderson BE, Casagrande JT, Rosario I, Gray GE. Oral contraceptive use and early abortion as risk factors for breast cancer in young women. *Br J Cancer* 1981; **43**: 72–76.

2. Ross RK, Paganini-Hill A, Gerkins VR, Mack TM, Pfeffer R, Arthur M, Henderson BE. A case-control study of menopausal estrogen therapy and breast cancer. *JAMA* 1980;**243**:1635-1639.

3. Thomas DB, Persing JP, Hutchinson WB. Exogenous estrogens and other risk factors for breast cancer in women with benign breast disease. *JNCI* 1982; **243**:1635-1639.

4. Vessey M, Baron J, Doll R, McPherson K, Yeates D. Oral contraceptives and breast cancer: final report of an epidemiological study. *Br J Cancer* 1983; **47**:455-462.

5. Brinton LA, Hoover R, Fraumeni JF Jr. Reproductive factors in the aetiology of breast cancer. *Br J Cancer* 1983; **47**: 757–62.

6. Lê MG, Bachelot A, Doyon F, Kramar A, Hill C. Oral contraceptive use and breast cancer or cervical cancer. Preliminary results of a French case-control study. In: Hormones and Sexual factors in Human Cancer Aetiology. Amsterdam: Elsevier Sci Publ, 1984.

7. Hislop TG, Coldman AJ, Elwood JM, Skippen DH, Kan L. Relationship between risk factors for breast cancer and hormonal status. *Int J Epidemiol* 1984; **15**: 469–76.

8. Hiatt RA, Bawol R, Friedman GD, Hoover R. Exogenous estrogen and breast cancer after bilateral oophorectomy. *Cancer* 1984; **54**: 139-144.

9. Nomura AMY, Kolonel LN, Hirohata T, Lee J. The association of replacement oestrogens with breast cancer. *Int J Cancer* 1986; **37**:49-53.

10. Lubin F, Wax Y, Modan B. Role of fat, animal protein and dietary fibre in breast cancer aetiology – a case-control study. *J Natl Cancer Inst* 1986; **77**: 605–12.

11. Lee NC, Rosero-Bixby L, Oberle MW, Grimaldo C, Whately AS, Rovira EZ. A case-control study of breast cancer and hormonal contraception in Costa Rica. *JNCI* 1987; **79**:1247-1254.

12. Ernster VL, Wrensch MR, Petrakis NL, King EB, Miike R, Murai T, Goodson WH, Siiteri PK. Benign and malignant breast disease: initial study results of serum and breast fluid analyses of endogenous estrogens. *JNCI* 1987; **79**: 949-60.

13. McPherson K, Vessey MP, Neil A, Doll R, Jones L, Roberts M. Early oral contraceptive use and breast cancer: results of another case-control study. *Br J Cancer* 1987; **56**: 653–60.

14. Alexander FE, Roberts MM, Huggins A. Risk factors for breast cancer with applications to selection for the prevalence screen. *J Epidemiol Comm Health* 1987;**41**:101-106.

15. Ewertz M, Duffy SW. Risk of breast cancer in relation to reproductive factors in Denmark. *Br J Cancer* 1988; **58**: 99–104.

16. Ravnihar B, Primic Zakelj M, Kosmelj K, Stare J. A case-control study of breast cancer in relation to oral contraceptive use in Slovenia. *Neoplasma* 1988; **35**: 109–21.

17. Rohan TE, McMichael AJ. Oral contraceptive agents and breast cancer: a population-based case-control study. *Med J Aust* 1988; **149**: 520–26.

18. Marubini E, Decarli A, Costa A, et al. The relationship of dietary intake and serum levels of retinol and beta-carotene with breast cancer. Results of a case-control study. *Cancer* 1988; **61**:173-80.

19. Yuan J-M, Yu MC, Ross RK, Gao Y-T, Henderson BE. Risk factors for breast cancer in Chinese women in Shanghai. *Cancer Research* 1988; **48**: 1949–53.

20. Siskind V, Schofield F, Rice D, Bain C. Breast cancer and breast feeding: results from an Australian case-control study. *Am J Epidemiol* 1989; **130**:229-236.

21. UK National Case-Control Study Group. Oral contraceptive use and breast cancer risk in young women. *Lancet* 1989; **333**: 973–82.

22. Bergkvist L, Adami H-O, Persson I, Hoover R, Schairer C. The role of breast cancer after estrogen and estrogen-progestin replacement. *N Engl J Med* 1989; **321**: 293-297.

23. Mills PK, Beeson WL, Phillips RL, Fraser GE. Prospective study of exogenous hormone use and breast cancer in seventh-day Adventists. *Cancer* 1989; **64**:591-597.

24. Adami HO, Bergstrom R, Lund E, Meirik O. Absence of and association between reproductive variables and risk of breast cancer in young women in Sweden and Norway. *Br J Cancer* 1990: **62**: 122–26.

25. Mettlin C, Croghan I, Natarajan N, Lane W. The association of age and familial risk in a case-control study of breast cancer. *Am J Epidemiol* 1990; **131**:973-83.

26. Schildkraut JM, Hulka BS, Wilkinson WE. Oral contraceptives and breast cancer: A case-control study with hospital and community controls. *Obstet Gynecol* 1990; **76**: 395-402.

27. Clavel F, Andrieu N, Gairard B, Bremond A, Piana L, Lansac J, Breart G, Rumeau-Rouquette C, Flamant R, Renaud R. Oral contraceptives and breast cancer: A French case-control study. *Int J Epidemiol* 1991: **20**: 32–38.

28. Kaufman DW, Palmer JR, de Mouzon J, Rosenberg L, Stolley PD, Warshauer ME, Zauber AG, Shapiro S. Am Estrogen replacement therapy and the risk of breast cancer: results from the case-control surveillance study. *J Epidemiol* 1991;**134**:1375-85.

29. Parazzini F La Vecchia C, Negri E. Spontaneous and induced abortion and risk of breast cancer. *Int J Cancer* 1991; **48**: 816–20.

30. Weinstein AL, Mahoney MC, Nasca PC, Leske MC. Varma AO. Breast cancer risk and oral contraceptive use: results from a large case-control study. *Epidemiology* 1991; **2**: 353–58.

31. Wingo PA, Lee NC, Ory HW, Beral V, Peterson HB, Rhodes P. Age-specific differences in the relationship between oral contraceptive use and breast cancer. *Obstet Gynecol* 1991; **78**: 161–70.

32. Segala C, Gerber M, Richardson S. The pattern of risk factors for breast cancer in a Southern France population. Interest for a stratified analysis by age at diagnosis. *Br J Cancer* 1991; **64**: 919-925.

33. Gajalakshami CK, Shanta V. Risk factors for female breast cancer: a hospital based case-control study in Madras, India. *Acta Oncologica* 1991; **30**:569-574.

34. Graham S, Hellmann R, Marshall J, Freudenheim J, Vena J, Swanson M, Zielezny M, Nemoto T, Stubbe N, Raimondo T. Nutritional epidemiology of postmenopausal breast cancer in western New York. *Am J Epidemiol* 1991;**134**:552-66.

35. Lee HP, Gourley L, Duffy SW, Esteve J, Lee J, Day NE. Risk factors for breast cancer by age and menopausal status: a case-control study in Singapore. *Cancer Causes Control* 1992; **3**: 313-322.

36. Miller AB, Baines CJ, To T, Wall C. Canadian National Breast Screening Study. I. Breast cancer detection and death rates among women aged 40-49 years. *Can Med Assoc J* 1992; **147**:1459-1476.

37. Rosenberg L, Palmer JR, Clarke EA, Shapiro S. A case-control study of the risk of breast cancer in relation to oral contraceptive use. *Am J Epidemiol* 1992; **136**: 1437–44.

38. Ursin G, Aragaki CC, Paganini-Hill A et al. Oral contraceptives and premenopausal bilateral breast cancer: a case-control study. *Epidemiology* 1992: **5**:414-9.

39. Wang Q-S, Ross RK, Yu MC, Ning J-P, Henderson BE, Kimm HT. A case-control study of breast cancer in Tianjin, China. *Cancer Epidemiology, Biomarkers Prevention* 1992; **1**: 435–39.

40. Yang CP, Daling JR, Band PR, Gallagher RP, White E, Weiss NS. Non-contraceptive hormone use and risk of breast cancer. *Cancer, Causes Control* 1992; **3**: 475–79.

41. Morabia A, Szklo M, Stewart W, Schuman L, Thomas DB. Consistent lack of association between breast cancer and oral contraceptives using either hospital or neighbourhood controls. *Preventive Med* 1993; **22**: 178–86.

42. Thomas DB, Noonan EA and the WHO Collaborative Study of Neoplasia and Steroid Contraceptives. Breast cancer and prolonged lactation. *Int J Epidemiology* 1993; **22**: 619–26.

43. Kalache A, Maguire A, Thompson SG. Age at last full-term pregnancy and risk of breast cancer. *Lancet* 1993; **341**:33-36.

44. Ngelangel CA, Lacaya LB, Cordero C, Laudico AV. Risk factors for breast cancer among Fillipino women. *Phil J Internal Medicine* 1994; **32**: 231-236.

45. Garrett PA, Hulka BS, Kim YL, Farber RA. HRAS photooncogene polymorphism and breast cancer. *Cancer Epidemiology, Biomarkers & Prevention* 1994; **2**:131-138.

46. Schairer C, Byrne C, Keyl PM, Brinton LA, Sturgeon SR, Hoover RN. Menopausal estrogen and oestrogen-progestin replacement therapy and risk of breast cancer (United States). *Cancer Causes Control* 1994; **5**: 491-500.

47. Daling JR, Malone KE, Voigt LF, White E, Weiss NS. Risk of breast cancer among young women: relationship to induced abortion. *J Natl Cancer Inst* 1994; **86**: 1584–92.

48. Lipworth L, Katsouyanni K, Ekbolm A, Michels KB, Trichopoulos D. Abortion and the risk of breast cancer: a case-control study in Greece. *Int J Cancer* 1995; **61**: 181–84.

49. Primic-Zakelj M, Evstifeeva T, Ravnihar B, Boyle P. Breast cancer risk and oral contraceptive use in Slovenian women aged 25 to 54. *Int J Cancer* 1995; **62**: 414–20.

50. Schuurman AG, van den Brandt PA, Goldbohm RA. Exogenous hormones and the risk of postmenopausal breast cancer. Results from the Netherlands cohort study. *Cancer Causes Control* 1995; **6**: 416-424.

51. La Vecchia C, Negri E, Franceschi S, Talamini R, Amadori D, Filiberti R, Conti E, Montella M, Veronesi A, Parazzini F, Ferraroni M, Decarli A. Oral contraceptives and breast cancer: a cooperative Italian study. *Int J Cancer* 1995; **60**:163-167.

52. Levi F, Lucchini F, Pasche C, La Vecchia C.Oral contraceptives, menopausal hormone replacement therapy and breast cancer risk. *Eur J Cancer Prev* 1996 ;**5** :259-66.

53. Viladiu P, Izquierdo A, de Sanjose S, Bosch FX. A breast cancer case-control study in Girona, Spain. Endocrine, familial and lifestyle factors. *Eur J Cancer* 1996;**5**:329-335.

54. Morabia A, Bernstein M, Heritier S, Khatchatrian N. Relation of breast cancer with passive and active exposure to tobacco smoke. *Am J Epidemiol* 1996;**143**:918-928.

55. Newcomb PA, Storer BE, Longnecker MP, Mittendorf R, Greenberg ER, Willett WC. Pregnancy termination in relation to induced abortion. *J Am Med Assoc* 1996; **275**: 283–87.

56. Rookus MA, van Leeuwen FE. Induced abortion and abortion and risk for breast cancer: reporting (recall) bias in a Dutch case-control study. *J Natl Cancer Inst* 1996; **88**: 1759–64.

57. Rossing MA, Stanford JL, Weiss NS, Habel LA. Oral contraceptive use and risk of breast cancer in middle-aged women. *Am J Epidemiol* 1996; **144**: 161–64.

58. Tavani A, La Vecchia C, Franceschi S, Negri E, D'avanzo B, Decarli A. Abortion and breast cancer risk. *Int J Cancer* 1996; **65**: 401–05.

59. Wu AH, Ziegler RG, Pike MC, et al. Menstrual and reproductive factors and risk of breast cancer in Asian-Americans. *Br J Cancer* 1996;**73**:680-6.

60. Brinton LA, Gammon MD, Malone KE, Schoenberg JB, Daling JR, Coates RJ. Modification of oral contraceptive relationships on breast cancer risk by selected factors among younger women. *Contraception* 1997; **55**:197-203.

61. Thomas HV, Key TJ, Allen DS, Moore JW, Dowsett M, I. Fentiman IS, Wang DY. Reversal of relation between body mass and edogenous estrogen concentrations with menopausal status. *J Natl Cancer Inst* 1997; **89**: 396–97.

62. McCredie MRE, Dite GS, Giles GG, Hopper JL. Breast cancer in Australian women under the age 40. *Cancer Causes Control* 1998; **9**: 189–98.

63. McCredie M, Paul C, Skegg DC, Williams S. Reproductive factors and breast cancer in New Zealand. *Int J Cancer* 1998; **76**: 182–88.

64. Ursin G, Ross RK, Sullivan-Halley J, Hanisch R, Henderson B, Bernstein L. Use of oral contraceptives and risk of breast cancer in young women. *Breast Cancer Res Treat* 1998;**50**:175-84.

65. Hopper JL, Chenevix-Trench G, Jolley D, et al. Design and analysis issues in a population-based case-control-family study of the genetic epidemiology of breast cancer, and the Co-operative Family Registry for Breast Cancer Families. *Monographs of the National Cancer Institute* 1999; **26**: 95–100.

66. Hirose K, Tajima K, Hamajima N, Kuroishi T, Kuzuya K, Miura S, Tokudome S. Comparative case-referent study of risk factors among hormone-related female cancers in Japan. *Jpn J Cancer Res* 1999; **90**:255-261.

67. Key T, Sharp GB, Appleby PN, Beral V, Goodman MT, Soda M, Mabuchi K. Soya food and breast cancer risk: a prospective study in Hiroshima and Nagasaki, Japan. *Brit J Cancer* 1999; **81**:1248-1256

68. Magnusson C, Baron JA, Correia N, Bergstrom R, Adami H-O, Persson I. Breast cancer risk following long-term oestrogen- and oestrogen-progestin-replacement therapy. *Int J Cancer* 1999; **81**:339-344.

69. Marcus PM, Baird DD, Millikan RC, Moorman PG, Qaqish B, Newman B. Adolescent reproductive events and subsequent breast cancer risk. *Am J Public Health* 1999; **89**: 1244–47.

70. Monninkhof EM, van der Schouw YT, Peeters PH. Early age at menopause and breast cancer: are leaner women more protected? A prospective analysis of the Dutch DOM cohort. *Breast Cancer Res Treat* 1999;**55**: 285-91

71. Urban M, Banks E, Egger S, Canfell K, O'Connell D, Beral V, Sitas F. Injectable and oral contraceptive use and cancers of the breast, cervix, ovary, and endometrium in black South African women: case-control study. *PLoS Med* 2012;**9(3)**: e1001182. doi:10.1371/journal.pmed.1001182

72. Chie W-C, Hseih C-C, Newcomb PA, et al. Age at any full term pregnancy and breast cancer risk. *Am J Epidemiol* 2000; **151**: 715-22.

73. Johnson KC, Hu J, Mao Y and the Canadian Cancer Registries Epidemiology Research Group. Passive and active smoking and breast cancer risk in Canada, 1994-97. *Cancer Causes Control* 2000; **11**:211-21.

74. Lazovich DA, Thompson JA, Mink PJ, Sellers TA, Anderson KE. Induced abortion and breast cancer risk. *Epidemiology* 2000; **11**: 76–80.

75. Newcomb PA, Mandelson MT. A record-based evaluation of induced abortion and cancer risk (United States). *Cancer Causes Control* 2000; **11**: 777–81.

76. Ross R K, Paganini-Hill A, Wan P C, Pike M C. Effect of Hormone Replacement Therapy on Breast Cancer Risk: Estrogen Versus Estrogen Plus Progestin. *Journal of the National Cancer Institute* 2000; **92**: 328-332.

77. Trentham-Dietz A, Newcomb PA, Egan KM, Titus-Ernstoff L, Baron JA, Storer BE, Stampfer M, Willett WC. Weight change and risk of postmenopausal breast cancer (United States). *Cancer Causes Control* 2000;**11**:533-42

78. Friedenreich CM, Bryant HE, Courneya KS. Case-Control Study of Lifetime Total Physical Activity and Breast Cancer Risk. *Am J Epidemiol* 2001;**154**:336 347.

79. Sanderson M, Shu XO, Jin F, Dai Q, Wanqing Wen WQ, Hai Y, Gao Y-T, Zheng W. Abortion history and breast cancer risk: results from the Shanghai Breast Cancer Study. *Int J Cancer* 2001; **92**: 889–905.

80. Tryggvadottir L, Tulinus H, Eyfjord JE, Sigurvinsson T. Breastfeeding and reduced risk of breast cancer in an Icelandic cohort study on breast cancer. *Am J Epidemiol* 2001; **154**: 37-42.

81. Wrensch MR, Petrakis NL, Miike R, et al. Breast cancer in women with abnormal cytology in nipple aspirates of breast fluid. *J Natl Cancer Inst* 2001; **93** :1791-8.

82. Calle EE, Rodriguez C, Jacobs EJ, et al. The American Cancer Society Prevention Study II Nutrition Cohort: rationale, study design, and baseline characteristics. *Cancer* 2002; **94** : 2490-2501.

83. Kirsch V, Kreiger N. Estrogen and estrogen-progestin replacement therapy and risk of postmenopausal breast cancer in Canada. *Cancer Causes Control* 2002;**13**:583-590.

84. Lumachi F, Ermani M, Brandes AA, Basso U, Paris M, Basso SM, Boccagni P. Breast cancer risk in healthy and symptomatic women: results of a multivariate analysis. A case-control study. *Biomed Pharmacother* 2002; **56**: 416-20.

85. Marchbanks PA, McDonald JA, Wilson HG, et al. The NICHD Women's Contraceptive and Reproductive Experiences Study: methods and operational results. *Ann Epidemiol* 2002;**12**:213-221.

86. Anderson GL, Manson J, Wallace R, Lund B, Hall D, Davis S, Shumaker S, Wang CY, Stein E, Prentice RL. Implementation of the Women's Health Initiative study design. *Ann Epidemiol* 2003 (9 Suppl):S5-17.

87. Becher H, Schmidt S, Chang-Claude J. Reproductive factors and familial predisposition for breast cancer by age 50. A case-control-family study for assessing the main effects and possible gene-environment interaction. *Int J Epidemiology* 2003; **32**: 38–48.

88. Ye Z, Gao DL, Qin Q, Ray RM, Thomas DB. Breast cancer in relation to induced abortions in a cohort of Chinese women. *Br J Cancer* 2002; **87**: 977–81.

89. Calle EE, Rodriguez C, Walker-Thurmond K, Thun MJ. Overweight, obesity, and mortality from cancer in a prospectively studied cohort of U.S. adults. *N Engl J Med* 2003; **348**:1625-38.

90. Dumeaux V, Alsaker E, Lund E. Breast cancer and specific types of hormonal contraceptives: a large Norwegian study. *Int J Cancer* 2003; **105**: 844-50.

91. John EM, Horn-Ross PL, Koo J. Lifetime physical activity and breast cancer risk in a multiethnic population: the San Francisco Bay Area breast cancer study. *Cancer Epidemiol Biomarkers Prev* 2003; **12**:1143-1152.

92. Olsson HL, Ingvar C, Bladstrom A. Hormone replacement therapy containing progestins and given continuously increases breast carcinoma risk in Sweden. *Cancer* 2003; **97**: 1387-1392.

93. Siquardson AJ, Doody MM, Rao RS et al. Cancer incidence in the US Radiologic Technologists Health Study, 1983-1988. *Cancer* 2003;**97**:3080-9.

94. Braaten T, Weiderpasse E, Kumle M, Adami H-O, Lund E. Education and risk of breast cancer in the Norwegian-Swedish Women's lifestyle and health cohort study. *Int J Cancer* 2004; **110**: 579-83.

95. Han DF, Ma J, Zhou X, Qiu H, Fang L, Huang S. A case-control study on the risk of female breast cancer in Wuhan area. *Zhonghua Liu Xing Bing Xue Za Zhi* 2004;**25**:256-60.

96. Stahlberg C, Pedersen AT, Lynge E, Andersen ZJ, Keiding N, Hundrup YA et al. Increased risk of breast cancer following different regimens of hormone replacement therapy frequently used in Europe. *Int J Cancer* 2004; **109**: 721-7.

97. Magnusson CM, Roddam AW, Pike MC, Chilvers C, Crossley B, Hermon C, McPherson K, Peto J, Vessey M, Beral V. Body fatness and physical activity at young ages and the risk of breast cancer in premenopausal women. *Br J Cancer* 2005;**93**:817-24.

98. Cauley JA, Lucas FL, Kuller LH, Vogt MT, Browner WS, Cummings SR. Bone mineral density and risk of breast cancer in older women: the study of osteoporotic fractures.Study of Osteoporotic Fractures Research Group. *JAMA* 1996; **276**:1404-8.

99. Lee S, Kolonel L, Wilkens L, et al. Postmenopausal hormone therapy and breast cancer risk: The Multiethnic Cohort *Int J Cancer* 2006;**118**:285-1291 .

100. Li CI, Malone KE, Daling JR. Interactions between body mass index and hormone therapy and postmenopausal breast cancer risk (United States). *Cancer Causes Control* 2006; **17**: 695-703.

101. Park SK, Garcia-Closas M, Lissowska J, et al. Intrauterine environment and breast cancer risk in a population-based case-control study in Poland. *Int J Cancer* 2006;**119**:2136-41.

102. Suzuki R, Rylander-Rudqvist T, Ye W, Saji S, Wolk A. Body weight and postmenopausal breast cancer risk defined by estrogen and progesterone receptor status among Swedish women: A prospective cohort study. *Int J Cancer* 2006; **119**:1683-9.

103. Rebbeck TR, Troxel AB, Norman S, Bunin G, DeMichele A, Schinnar R, Berlin JA, Strom BL. Pharmacogenetic modulation of combined hormone replacement therapy by progesterone-metabolism genotypes in postmenopausal breast cancer risk. *Am J Epidemiol* 2007;166:1392-9.

104. Roddam AW, Pirie K, Pike MC, et al. Active and passive smoking and the risk of breast cancer in women aged 36-45 years: a population based case-control study in the UK. *Br J Cancer* 2007; **97**:434-9.

105. Shantakumar S, Terry MB, Paykin A, et al. Long Island: Age and menopausal effects of hormonal birth control and hormone replacement therapy in relation to breast cancer risk. *Amer J Epidemiol* 2007;**165**:1187-1198.

106. Brinton LA, Richesson D, Leitzmann MF, et al. Menopausal Hormone Therapy and Breast Cancer Risk in the NIH-AARP Diet and Health Study Cohort. *Cancer Epi Biomarkers Prev* 2008:**17**;3150-3160.

107. Fiesch-Janys D, Slanger T, Mutschelknauss E, et al. Risk of different histological types of postmenopausal breast cancer by type and regimen of menopausal hormone therapy. *Int J Cancer* 2008;**123**:933-941.

108. Bakken K, Fournier A, Lund E, et al. Menopausal hormone therapy and breast cancer risk: impact of different treatments.The European Prospective Investigation into Cancer and Nutrition. *Int J Cancer* 2010;**128**:144-156.

109. Beasley JM, Coronado GD, Livaudais J, et al. Alcohol and risk of breast cancer in Mexican women. *Cancer Causes Control* 2010; **6**:863-70.

110. Hadjisavvas A, Loizidou MA, Middleton N, et al. An investigation of breast cancer risk factors in Cyprus: a case control study. *BMC Cancer* 2010; **10**:447.

111. Hunter DJ, Colditz GA, Hankinson SE, Malspeis S, Spiegelman D, Chen W, Stampfer MJ, Willett WC. Oral contraceptive use and breast cancer: a prospective study of young women. *Cancer Epidemiol Biomarkers Prev* 2010;**10**:2496-502.

112. Kawai M, Minami Y, Kuriyama S, et al.Reproductive factors, exogenous female hormone use and breast cancer risk in Japanese: the Miyagi Cohort Study. *Cancer Causes Control* 2010;**21**:135-45.

113. Chen WY, Rosner B, Hankinson SE, Colditz GA, Willett WC. Moderate alcohol consumption during adult life, drinking patterns, and breast cancer risk. *JAMA* 2011;**306**:1884-90.

114. Beral V, Reeves G, Bull D, Green J, for the Million Women Study Collaborators. Breast cancer risk in relation to the interval between menopause and starting hormone therapy. *J Natl Cancer Inst* 2011;**103**:296–305

115. Ghiasvand R, Maram ES, Tahmasebi S, Tabatabaee SH. Risk factors for breast cancer among young women in southern Iran. *Int J Cancer* 2011;**129**:1443-9.

116. Suzuki R, Iwasaki M, Inoue M, Sasazuki S, Sawada N, Yamaji T, Shimazu T, Tsugane S; Japan Public Health Center-based Prospective Study Group. Body weight at age 20 years, subsequent weight change and breast cancer risk defined by estrogen and progesterone receptor status--the Japan public health center-based prospective study. *Int J Cancer* 2011;**129**:1214-24.

117. Sharma A, Gentry-Maharaj A, Burnell M, Fourkala EO, Campbell S, Amso N, Seif MW, Ryan A, Parmar M, Jacobs I, Menon U; UK Collaborative Trial of Ovarian Cancer Screening (UKCTOCS). Assessing the malignant potential of ovarian inclusion cysts in postmenopausal women within the UK Collaborative Trial of Ovarian Cancer Screening (UKCTOCS): a prospective cohort study. *BJOG* 2012;**119**:207-19.
